# Enhancing tyrosine kinase inhibitor sensitivity by restoring IKAROS activity on GLUT1 expression and glycolysis in Philadelphia chromosome-positive acute lymphoblastic leukemia

**DOI:** 10.1038/s41375-026-02898-2

**Published:** 2026-03-06

**Authors:** Linyao Zhang, Qi Han, Huimin Xiang, Rosa Lapalombella, Ann-Kathrin Eisfeld, Walter G. Hanel, Jonathan E. Brammer, Alice S. Mims, Jennifer A. Woyach, Chunhua Song, Zheng Ge

**Affiliations:** 1 Department of Hematology, Zhongda Hospital, School of Medicine, Southeast University, Institute of Hematology Southeast University, Nanjing, China; 2 Division of Hematology, The Ohio State University Wexner Medical Center, The James Cancer Hospital, Columbus, OH USA

**Keywords:** Translational research, Cancer metabolism, Cell signalling

## Abstract

Many patients with Philadelphia chromosome-positive acute lymphoblastic leukemia (Ph+ ALL) are still less sensitive to tyrosine kinase inhibitors (TKIs). Ph+ ALL shows a high incidence of *IKZF1* deletions. Casein kinase II (CK2)-mediated hyperphosphorylation of *IKZF1*, encoding protein IKAROS, contributes to its dysfunction, and CK2 inhibitor, CX-4945, restores IKAROS function in high-risk ALL. Here, we found that Ph+ ALL cells with *IKZF1* deletion are inherently resistant to TKIs. The combination of TKIs (imatinib or ponatinib) with CX-4945 significantly extended the survival and reduced the tumor burden in the *IKZF1* deletion (Ik6^+^) Ph+ ALL patient-derived xenograft (PDX) mouse model; particularly, the patient died of relapse shortly after treatment with the third-generation TKI and the CD19/CD3 bispecific antibody blinatumomab. *GLUT1* is highly expressed in the Ph+ ALL and associated with synergy of TKIs with CX-4945; Seahorse assay showed enhanced glycolysis in the patient sample with Ik6^+^ Ph+ ALL; *GLUT1* knockdown suppresses glycolysis and induces apoptosis in the cells. The combination of TKIs with CX-4945 demonstrates the synergistic efficacy through restoring IKAROS transcriptional repression of *GLUT1* and further suppressing glycolysis in Ph+ ALL. Our results identify new mechanisms underlying TKI sensitivity and novel approaches to overcome TKI resistance through transcriptional repression of the key genes in glycolysis in Ph+ ALL.

## Introduction

Acute lymphoblastic leukemia (ALL) is an aggressive clonal hematologic cancer of lymphoid progenitor cells residing within the bone marrow [1]. The Philadelphia (Ph) chromosome-positive ALL (Ph+ ALL) is characterized by the [t (9;22) (q34; q11)] translocation, i.e., the *BCR::ABL1* fusion protein, which appears in about 3–5% of children and 20–30% of adults with ALL.

Adult Ph+ ALL has an inferior prognosis [2–6], although the outcomes of pediatric Ph+ ALL have been revolutionized. Traditional chemotherapy for adult Ph+ ALL has limited efficacy [7]. Tyrosine kinase inhibitors (TKIs) dramatically improved the survival of Ph+ ALL from 25% to almost 80% [7–13]. The first TKI to be added to induction chemotherapy for ALL is imatinib [10, 14]; however, imatinib is no longer the first-line TKI of choice for Ph+ ALL due to its lower potency and higher risk of resistance, and second-generation and third-generation TKIs have now become the standard of care [15]. Nevertheless, despite the encouraging results of TKIs, many patients with Ph+ ALL have suboptimal responses. Even ponatinib, with its remarkable efficacy in the treatment of Ph+ ALL, still cannot eliminate the risk of relapse, particularly in high-risk patients [16–18]. Moreover, there are limited options for clinical therapies of TKI resistance Ph+ ALL, which include chimeric antigen receptor T (CAR-T), bispecific antibodies, and stem cell transplantation [19–22]. Therefore, it is crucial to comprehend the molecular mechanisms underlying TKI sensitivity and devise novel therapeutic strategies to counteract drug resistance in Ph+ ALL.

Glycolysis plays a key role in cancer cell growth and development. Cancer cells rely on glycolysis to produce energy, even when oxygen is available. Glycolysis also contributes to the development of chemoresistance [23–25]. The underlying molecular mechanisms linking glycolysis to chemoresistance of TKI therapy in B-cell ALL (B-ALL) remain to be defined.

*IKZF1* encoding protein IKAROS is a lymphoid transcription factor and functions as a tumor suppressor in ALL [26, 27]. The *IKZF1* genetic defect is a hallmark of ALL. Ph+ ALL shows a high incidence of *IKZF1* deletions, with approximately two-thirds of Ph+ ALL cases having an *IKZF1* deletion/mutations. IKAROS isoform 6 (Ik6) is an exon 4–7 deletion mutant of the *IKZF1* gene, which is most frequently detected in Ph+ ALL patients, reaching 83.7% in adults [28–30]. Moreover, the blinatumomab, CAR-T, or allogeneic hematopoietic stem cell transplantation (allo-HSCT) significantly improves the outcome of the Ph+ ALL [31–33]. However, the Ph+ ALL patients with *IKZF1*-plus signature have inferior survival outcomes with shorter relapse-free survival [34–36]. While ~60% of patients with *IKZF1* deletion/mutations exhibit the *IKZF1*-plus signature [34, 37, 38]. Recent reports have shown that the *IKZF1* genetic defect is associated with resistance to conventional chemotherapy and targeted therapy [39–43]. *IKZF1* deletion confers resistance to CAR19 in relapsed/refractory B-ALL (r/r B-ALL) via CD19 intron retention [44]; and two of 27 Ph+ ALL patients with *IKZF1* deletion relapsed, after receiving treatment of second-generation TKI and CAR-T [45]. Also, IKAROS function is associated with allo-HSCT [46, 47], and leukemia cells with low levels of IKAROS can become resistant to blinatumomab and other CD19-targeted therapies [48].

Beyond the genetic defects in *IKZF1*, we reported that casein kinase II (CK2)-mediated hyperphosphorylation of IKAROS contributes to its dysfunction in high-risk ALL, and CK2 inhibitor CX-4945 restores IKAROS function and shows therapeutic potential in ALL [40, 41, 43, 49]. Moreover, although all *IKZF1* deletion/mutations are heterozygous [50–52], CX-4945 demonstrated comparable therapeutic efficacy in patient samples with *IKZF1* deletion/mutations as that of *IKZF1* wild-type (WT) [40, 43, 53–55].

In the present study, we found less sensitivity of SUP-B15 cells and the primary samples from Ph+ ALL patients with Ik6 to TKIs (imatinib and ponatinib) compared to those without Ik6, and the TKI sensitivity is associated with high *GLUT1* expression and enhanced glycolysis in the cells. Our data showed that CX-4945 significantly enhances the therapeutic efficacy of TKIs in Ik6^+^ Ph+ ALL patient-derived xenograft (PDX) mouse model by repression of *GLUT1* transcription and suppression of glycolysis in the cells; particularly, this PDX model used the cells from the patient who relapsed and died 6 months after the treatment of third-generation TKI and the bispecific T-cell engager molecule blinatumomab (an anti-CD19 and anti-CD3 bispecific antibody). Our results identify new mechanisms underlying TKI sensitivity and novel approaches to enhance the drug sensitivity through transcriptional repression of the key genes in glycolysis in Ph+ ALL, which highlight the clinical significance of the combination of TKIs with CX-4945 in r/r B-ALL.

## Methods and materials

### Clinical samples and primary cells

All patients provided informed consent, and the study was approved by the Ethics Committee for Clinical Research of Zhongda Hospital Southeast University, and by the tenets of the Declaration of Helsinki. A total of 20 bone marrow (BM) samples from Ph+ ALL patients (Supplementary Table S1) and 20 normal control samples of mononuclear cells from healthy volunteers were collected from Zhongda Hospital, Southeast University. The mononuclear cells from primary samples of three Ph+ ALL patients (Supplementary Table S2) collected by leukapheresis were separated using a lymphocyte separation medium (MP Biomedicals, USA), and erythrocytes were lysed by red blood cell (RBC) lysis buffer (Biosharp, China). Patient 1 is a relapsed Ph+ ALL; Patient 2 is de novo Ik6^+^ Ph+ ALL, but this patient died of relapse shortly after the treatment of third-generation TKI and blinatumomab; Patient 3 is a de novo Ph+ ALL. The resulting cells from the patient samples are applied for in vitro molecular and cellular assays, and for in vitro culture with drug treatment.

### Cell lines and cell culture

The B-ALL cell line SUP-B15 was obtained from ATCC, USA. This cell line expresses the *BCR::ABL1* gene and its fusion protein P190 and also contains the *IKZF1* deletion, the Ik6 isoform. The cell line and primary cells were cultured in Roswell Park Memorial Institute 1640 medium (RPMI-1640, Gibco, China). All cell culture media contained 10% fetal bovine serum (FBS, Hyclone, China) and were cultured at 37 °C with a 5% CO_2_ atmosphere. Logarithmic growth phase cells were used for the experiment. Every two to three days, the medium was changed.

### Plasmid construction, lentiviral transduction, and target gene knockdown

*IKZF1* cDNA was subcloned into the lentiviral OE-RNA vector (pLV4ltr-PGK-ZsGreen(2A)Puro-CMV) (Corues Biotechnology, China). Lentiviral shRNA plasmids for *GLUT1* were constructed by subcloning the shRNA oligos into the lentiviral shRNA vector (pLV3ltr-ZsGreen-Puro-U6) (Corues Biotechnology, China) according to the manufacturer’s recommendations [40, 43]. Lentivirus was generated, and the cells were transduced as we previously reported [40, 41, 43, 49].

### Real-time quantitative Polymerase Chain Reaction (RT-qPCR) for patient samples

The Real-time quantitative Polymerase Chain Reaction (RT-qPCR) assay was performed as previously reported [40, 41, 43, 49]. The comparative Ct method was used to determine the relative expression levels of the target genes, which were reported as 2^−ΔCt^ for clinical samples. The characterization of the patients is shown in Supplementary Table S1. The primer sequences for RT-qPCR are listed in Supplementary Table S3.

### Glucose consumption and lactic acid production assay

Cells were inoculated in 24-well plates at a 4 × 10^5^ cells/mL density and treated for 48 h. The culture medium supernatant was obtained for glucose consumption and lactic acid production, which were measured using a detection kit (Jiancheng, China) according to the manufacturer’s instructions. The absorbance at 505 nm of glucose consumption was measured using the microplate reader (Bio-Tek, USA). The lactic acid production absorbance at 530 nm. At least three independent assays were performed.

### Seahorse assay

The real-time changes in extracellular acidification rate (ECAR) were measured using the XF96 Seahorse Extracellular Flux Analyzer (Seahorse Bioscience, USA). Cells were plated in 6-well plates at a density of 4 × 10^5^ cells/mL in 6-well plates and treated with imatinib alone, ponatinib alone, CX-4945 alone, and imatinib or ponatinib with CX-4945 (Combo) for 48 h. The resulting cells were collected for the Seahorse assay. Briefly, the XF96-well cell culture microplate (Agilent, USA) was pre-coated with Cell-Tak, and the cells were re-inoculated and cultured. 2 mmol/L glutamine was added to the Seahorse XF RPMI base medium, and the pH of the solution was adjusted to 7.4 using NaOH. ECAR was simultaneously measured in basal conditions and after the subsequent addition of 10 mM glucose, 1 μM oligomycin, and 50 mM 2-deoxyglucose (2-DG). All measurements were taken following the manufacturer’s instructions and analyzed using Seahorse Wave software.

### Ik6^+^ Ph + ALL patient-derived xenograft (PDX) mouse model

The patient sample (Patient 2, Supplementary Table S2) for the PDX mouse model was obtained from Zhongda Hospital, Southeast University. To get enough cells for the PDX mouse model, the patient’s BM samples were amplified once in the female *NOD/ShiLtJGpt-Prkdc*^*em26Cd52*^*Il2rg*^*em26Cd22*^*/Gpt* (NCG) mice. The amplified samples were validated to be consistent with the original patient sample by immunophenotyping and next-generation sequencing. Various doses of the cells (0.5 × 10^7^ to 2 × 10^7^ per mouse) were transplanted intravenously into NCG mice (GemPharmatech Co., Ltd, Nanjing, China) for optimizing the doses, and 1 × 10^7^ Ph+ ALL cells per mouse were identified as the best dose for the PDX mouse model based on the death days.

Following engraftment, mice (*n* = 16 per group) received the treatment in six groups: Vehicle (Group1); Imatinib via intraperitoneal injection at 25 mg/kg/day, 5 times a week (Group 2); Ponatinib daily via gavage at 10 mg/kg/day (Group 3); CX-4945 daily via gavage at 100 mg/kg/day (Group 4), Combination treatment with imatinib and CX-4945 at the same doses as single drug groups (Group 5); and combination treatment with ponatinib and CX-4945 at the same doses as single drug groups (Group 6). Then, when the vehicle mice met the early removal criteria due to the excessive leukemia burden, 8 mice per group were euthanized to observe the drug efficacy on leukemia burden. The single-cell suspension of euthanized mice BM or spleen cells was collected, and the RBC were lysed. The resulting cells were used for living cell counts and flow cytometry analysis of leukemia burden. The remaining 8 mice per group were followed until the mice died or met early removal criteria for survival analysis. The dead mice were counted daily, and the Kaplan-Meier method was used to generate the survival curves and analyze the survival difference.

All experimental operations were performed with the consent of the Animal Care Committee of Southeast University and complied with the Regulations for the Administration of Affairs Concerning Experimental Animals of China.

Details and additional experimental methods are found in the Supplemental Materials.

## Results

### Comparing the sensitivity of Ph + ALL with or without Ik6 to TKIs

**Fig. 1:**
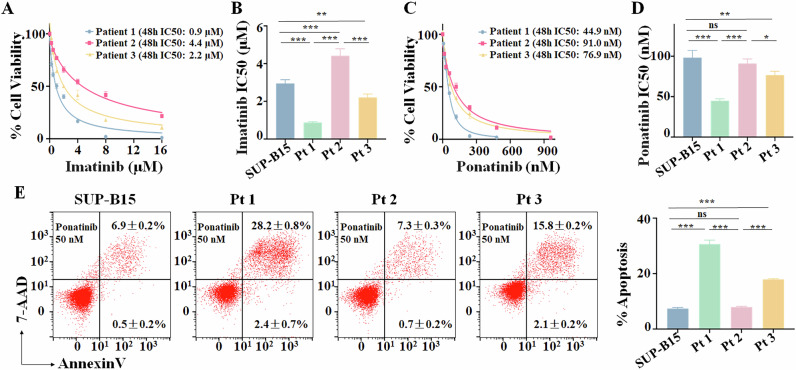
Comparison of imatinib or ponatinib sensitivity on cell proliferation arrest in SUP-B15 and primary cells. **A** Effect of imatinib on cell proliferation of primary cells from Ik6^+^ Ph+ ALL patients (Patient 2, Pt 2) or without *IKZF1* genetic defects [Patient 1 (Pt 1), Patient 3 (Pt 3)]. **B** Imatinib 50% inhibitory concentration (IC50) values for 48 h in SUP-B15 and primary cells were calculated, and a statistical histogram was observed. **C** Effect of ponatinib on cell proliferation of primary cells. **D** Ponatinib IC50 values for 48 h in SUP-B15 and primary cells were calculated, and a statistical histogram was observed. For **A**, **C**, primary cells were treated with various doses of imatinib or ponatinib for 48 h. **E** Effect of ponatinib on apoptosis of SUP-B15 and primary cells. The cells were treated with Pt 1’s IC50 doses of ponatinib (50 nM) for 48 h. *P < 0.05, **P < 0.01, ***P < 0.001, ns no significance.

In this study, SUP-B15 cells and three B-ALL patient samples were used. SUP-B15 cells and Patient 2 (Pt 2) samples express both the *BCR::ABL1* fusion gene and Ik6 (Ik6^+^ Ph+ ALL), and Pt 2 had a very poor outcome, even relapsed and died 6 months after the treatment of third-generation TKI and blinatumomab. While Patient 1 (Pt 1, relapse) and Patient 3 (Pt 3, de novo) samples express the *BCR::ABL1* fusion gene (Ph+ ALL) without *IKZF1* deletion. To observe the sensitivity to TKIs, these cells were treated with various doses of imatinib or ponatinib for 24, 48, or 72 h (Fig. 1A–D, Supplementary Fig. S1A, B). Both imatinib and ponatinib induced a dose-dependent and time-dependent proliferation arrest in SUP-B15 cells (Supplementary Fig. S1A, B), as well as a dose-dependent effect in the three patient samples (Fig. 1A, C). The 50% inhibitory concentration (IC50) values for imatinib and ponatinib are significantly higher in Ik6^+^ Ph+ ALL cells (SUP-B15 and Pt 2) *vs*. those without *IKZF1* deletion (Pt 1 and Pt 3) (Fig. 1B, D). These data indicate that Ph+ ALL cells with Ik6 are significantly less sensitive to imatinib and ponatinib than those without Ik6.

Cell cycle analysis and apoptosis showed that the G0/G1 phase arrest and % apoptosis were increased in a dose-dependent manner in SUP-B15 cells treated with IC50 and 2× IC50 imatinib or ponatinib for 48 h, and the increase is significant compared to vehicle control for cell cycle arrest (Supplementary Fig. S2A, C) and apoptosis (Supplementary Fig. S2B, D).

In addition, we compared the effect of imatinib or ponatinib on apoptosis in Ph+ ALL with Ik6 (SUP-B15 and Pt 2) *vs*. those without Ik6 (Pt 1 and Pt 3) upon the treatment with IC50 doses of the drugs for 48 h. Results showed that the % apoptosis in SUP-B15 cells and Pt 2 samples is significantly less than that in Pt 1 and Pt 3 samples (Fig. 1E, Supplementary Fig. S1C). These data indicated that Ph+ ALL cells with Ik6 are much less sensitive to imatinib or ponatinib than those without Ik6.

### Restoring IKAROS function leads to the anti-leukemia effect in Ph + ALL cells

As *IKZF1* deletion is common in Ph+ ALL, we explored the effect of overexpression of IKAROS on cell proliferation, cell cycle, and apoptosis of SUP-B15 cells. We confirmed the expression level of overexpressed IKAROS (OE-IKAROS) by Western blot (Supplementary Fig. S3A) and RT-qPCR (Supplementary Fig. S3B) compared to Vector-only control (Vector). Results showed that OE-IKAROS significantly induced cell proliferation inhibition (Supplementary Fig. S3C), G0/G1 phase arrest (Supplementary Fig. S3D), and apoptosis (Supplementary Fig. S3E) compared to the Vector-only control.

**Fig. 2:**
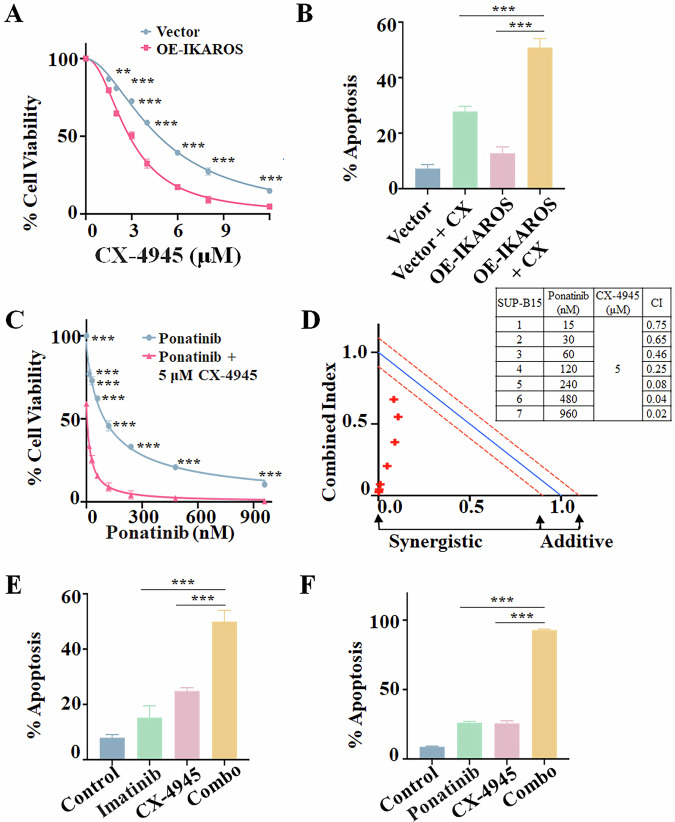
Restoring *IKZF1* function results in an anti-leukemia effect and the synergistic effect of imatinib or ponatinib with CX-4945 in SUP-B15 cells. **A** Comparing the effect of CX-4945 on cell proliferation in SUP-B15 cells with overexpressed IKAROS (OE-IKAROS) *vs*. Vector-only (Vector). **B** Comparing the effect of CX-4945 (CX) *vs*. Vehicle control on apoptosis in SUP-B15 cells with OE-IKAROS *vs*. Vector-only, i.e., in four groups, Vector+Vehicle (Vector), Vector+CX-4945, OE-IKAROS+Vehicle (OE-IKAROS), OE-IKAROS + CX-4945. The cells were treated with CX-4945 or Vehicle for 48 h. **C** Effect of the combination of ponatinib with CX-4945 on the cell proliferation arrest of SUP-B15 cells. The cells were treated with various doses of ponatinib with IC50 CX-4945 for 48 h. **D** CalcuSyn analysis of the combination of proliferation arrest in SUP-B15 cells. Effect of **E** imatinib or **F** ponatinib with CX-4945 on apoptosis in SUP-B15 cells compared to single drug control and Vehicle only control. For **E**, **F** the cells were treated with 3 μM imatinib or 100 nM ponatinib, 5 μM CX-4945, and the combination for 48 h. **P < 0.01, ***P < 0.001.

CK2 inhibitor CX-4945 can restore IKAROS function even in the *IKZF1* deletion/mutation B-ALL cells [40, 41, 49]. Then, we examine the effect of CX-4945 on the SUP-B15 cells. Results showed that CX-4945 inhibited the proliferation of SUP-B15 cells in a dose-dependent and time-dependent manner (Supplementary Fig. S4A). Also, CX-4945 significantly induced G0/G1 phase arrest (Supplementary Fig. S4B) and apoptosis (Supplementary Fig. S4C) in the cells. Moreover, CX-4945 treatment showed a significantly higher effect on cell proliferation inhibition (Fig. 2A), G0/G1 phase arrest (Supplementary Fig. S5A), and apoptosis (Fig. 2B, Supplementary Fig. S5B) in OE-IKAROS-expressing SUP-B15 cells *vs*. Vector-only control.

### CX-4945 sensitizes the effect of imatinib or ponatinib on the anti-tumor effect in Ph + ALL cells

To observe the synergistic effect of imatinib or ponatinib with CX-4945, SUP-B15 cells were treated with different doses of imatinib or ponatinib with various doses of CX-4945 for 48 h. Results showed that the combination of 5 μM (IC50) and 10 μM (2× IC50) CX-4945 with imatinib has significantly increased cell proliferation arrest compared to the single drug control (Supplementary Fig. S6A). Ponatinib with 5 μM CX-4945 has obviously increased cell proliferation arrest compared to the single drug control (Fig. 2C). CalcuSyn analysis also shows that the combination of imatinib with IC50 or 2× IC50 CX-4945, and ponatinib with IC50 CX-4945 have synergistic effects (Fig. 2D, Supplementary Fig. S6B–F). Furthermore, the Bliss independence model analysis also confirmed the synergistic effect of imatinib with 5 μM or 10 μM CX-4945 in SUP-B15 cells (Supplementary Fig. S6G). Consistently, the combination of CX-4945 with imatinib or ponatinib significantly induced G0/G1 phase arrest (Supplementary Fig. S7A, B) and apoptosis (Fig. 2E, F, Supplementary Fig. S7C, D) compared to the single-drug controls in the cells. The cell colony-forming assay showed that the combination of CX-4945 with imatinib or ponatinib has significantly lower colony numbers compared to either single drug control in the cells (Supplementary Fig. S7E, F). These results indicated that CX-4945 potentiates the inhibitory effect of TKIs in vitro.

### Synergistic effect of imatinib or ponatinib with CX-4945 on cell growth arrest and apoptosis in Ph + ALL patient samples

**Fig. 3:**
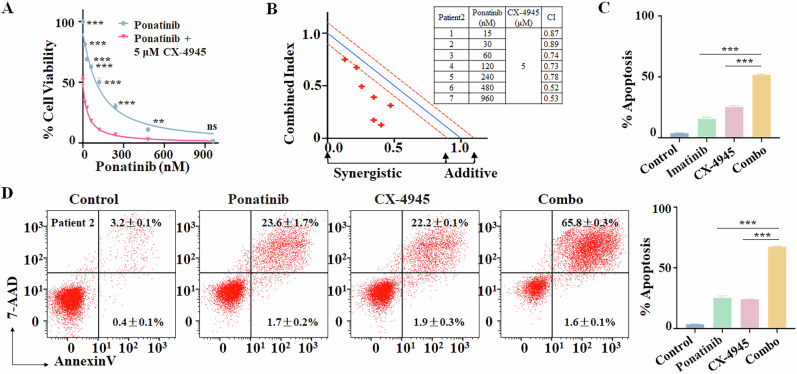
The synergistic effect of imatinib or ponatinib with CX-4945 in primary cells from the Ik6^+^ Ph + ALL patient (Pt 2). **A** Effect of the combination of ponatinib with CX-4945 on the cell proliferation arrest of Pt 2’s primary cell. The cells were treated with various doses of ponatinib with IC50 CX-4945 for 48 h. **B** CalcuSyn analysis of the combination of proliferation arrest in primary cells. Effect of **C** imatinib or **D** ponatinib with CX-4945 on apoptosis in primary cells compared to single drug control and Vehicle only control. For **C**, **D** the cells were treated with 4 μM imatinib or 100 nM ponatinib, 5 μM CX-4945, and the combination for 48 h. **P < 0.01, ***P < 0.001, ns no significance.

Further, we examined the effect of the combination of imatinib or ponatinib with CX-4945 in primary cells from the three Ph+ ALL patients (Pt 1-3). The IC50 of CX-4945 on cell growth arrest was determined for 48 h (Supplementary Figs. S8A, S9A, S10A). Results showed that the combination of imatinib or ponatinib with CX-4945 in the dose of either IC50 significantly increased the cell growth arrest compared to either single drug controls in primary cells of Pt 2 (Fig. 3A, Supplementary Fig. S9B), Pt 1 (Supplementary Fig. S8B, C), and Pt 3 (Supplementary Fig. S10B, C), and CalcuSyn analysis showed the combination of imatinib or ponatinib with the IC50 dose of CX-4945 had the synergistic effect in the three patient samples (Fig. 3B, Supplementary Figs. S8D, E, S9C, S10D, E). Similar results were observed for the effect of the combination on apoptosis for the three patient samples (Fig. 3C, D, Supplementary Figs. S8F, G, S9D, S10F, G). In addition, no significant difference was observed in cell proliferation arrest and apoptosis between the Ph+ ALL with or without Ik6 upon the combination treatment (data not shown). These data indicated that the combination of imatinib or ponatinib with CX-4945 has a synergistic effect on cell growth arrest and apoptosis in Ph+ ALL primary cells with or without Ik6, revealing the clinical application for Ph+ ALL therapy. These data also show that the combination of CX-4945 with TKIs appears to have a comparable effect on Ph+ ALL with or without Ik6, revealing that CX-4945 sensitizes the effect of TKIs and reverses the drug resistance in Ph+ ALL.

### The combination of TKIs with CX-4945 inhibits leukemia development of Ph + ALL in vivo

To assess the in vivo synergistic efficacy of imatinib or ponatinib combined with CX-4945, a human SUP-B15 leukemia cell-derived xenograft (CDX) and an Ik6^+^ Ph+ ALL PDX mouse model were developed by tail-vein injection of the cells into NCG mice.

**Fig. 4:**
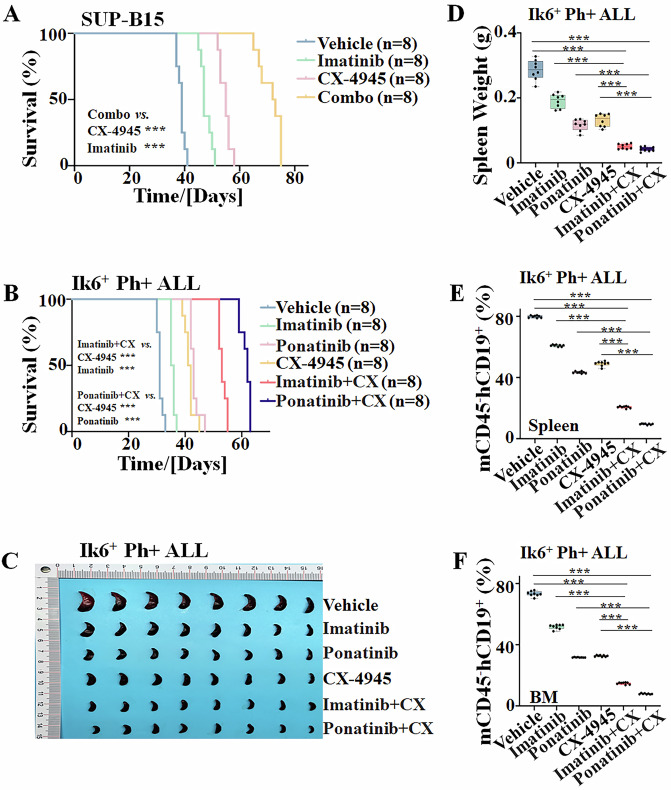
Synergistic efficacy of imatinib or ponatinib with CX-4945 on leukemia development in the SUP-B15-xenograft (CDX) and Ik6^+^ Ph + ALL patient-derived xenograft (PDX) mouse model. Comparison of Kaplan–Meier survival curves of the combination of imatinib with CX-4945 (CX) or ponatinib with CX-4945 *vs*. either single drug controls in **A** CDX and **B** PDX mouse model. **C** Comparison of spleen size of the six groups in PDX mouse model. **D** Comparison of spleen weight of the six groups in PDX mouse model. The representative quantitative data of mCD45^-^hCD19^+^ cells in the **E** spleen and **F** bone marrow (BM) of PDX mouse model. For **E**, **F** the mice were euthanized after the treatment scheme was completed, the spleens were isolated, and the splenocytes and BM cells were prepared. ***P < 0.001.

SUP-B15 (2 × 10^7^ cells/mouse) cells were intravenously injected into NCG mice. After the leukemia engraftment, the mice received a treatment scheme in four groups: Group 1 (Vehicle), Group 2 (imatinib alone), Group 3 (CX-4945 alone), and Group 4 (imatinib+CX-4945 combo) as shown in Supplementary Fig. S11A. Results indicated that the combination of imatinib with CX-4945 significantly increased the survival of the mouse model (Fig. 4A), decreased the spleen sizes (Supplementary Fig. S11B) and weights (Supplementary Fig. S11C) of the spleens, and decreased the percentage of mouse CD45^-^ human CD19^+^ Ph+ ALL cells in the spleen (Supplementary Fig. S11D) and BM (Supplementary Fig. S11E) compared to either single treatment controls.

For the PDX mouse model, the primary cells (1 × 10^7^ cells/mouse) from Ik6^+^ Ph+ ALL (Pt 2. Supplementary Table S2) were intravenously injected into NCG mice. Pt 2 had 90% blasts and 98% *BCR::ABL1* in BM at newly diagnosis, and had a very poor outcome, even treated with third-generation TKI and blinatumomab, with relapse-free survival (RFS) and overall survival (OS) was 6 months and 11 months, respectively. The leukemia engraftment was monitored before the treatment, and at the beginning of the treatment, it was confirmed that the proportion of leukemia blast cells in the peripheral blood of the mice was ≥5%. The treatment schemes for PDX are shown in Supplementary Fig. S12A. Results showed that the combination of imatinib or ponatinib with CX-4945 significantly prolonged the survival of the PDX models (Fig. 4B), decreased the spleen sizes (Fig. 4C) and weights (Fig. 4D), decreased the percentage of mouse CD45^-^ human CD19^+^ Ph+ ALL cells in the spleen (Fig. 4E, Supplementary Fig. S12B) and BM (Fig. 4F, Supplementary Fig. S12C) compared to either single treatment controls.

These data demonstrated that the combination of imatinib or ponatinib with CX-4945 has synergistic efficacy in both CDX and PDX mouse models.

### Roles of *GLUT1* and glycolysis in Ph + ALL

**Fig. 5:**
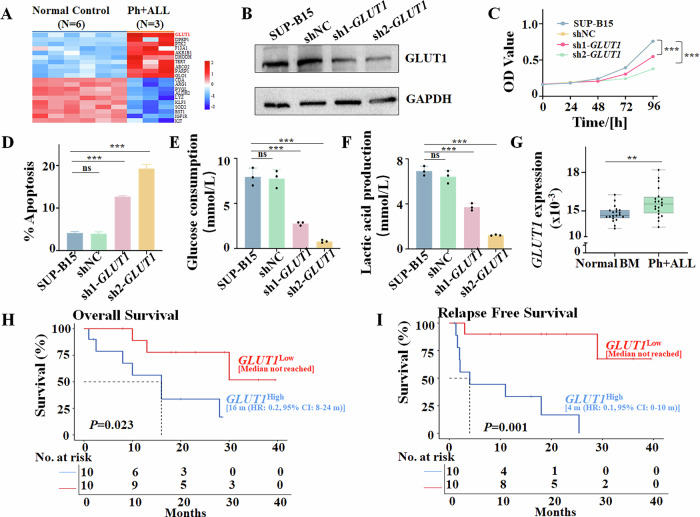
Roles of *GLUT1* in SUP-B15 cells and Ph + ALL. **A** Heatmap of the top overlapped DEGs. **B** Comparison of the *GLUT1* protein level in SUP-B15 cells with sh*GLUT1 vs*. shNC or SUP-B15, detected by Western blot. SUP-B15 cells were stably transduced with lentiviral *GLUT1* shRNA (sh*GLUT1*) or scramble shRNA (shNC). Comparison of (**C**) cell viability effect and **D** apoptosis in SUP-B15 cells with sh*GLUT1 vs*. shNC or SUP-B15. Comparison of **E** glucose consumption and **F** lactic acid production in SUP-B15 cells with sh*GLUT1 vs*. shNC or SUP-B15. The stable transduced sh*GLUT1* or shNC SUP-B15 cells were inoculated in 24-well plates at 4 × 10^5^ cells/mL. Then, cells were treated for 48 h. The supernatant of the cells was collected for detection with the kits following the manual. **G** Comparison of *GLUT1* mRNA levels in Ph+ ALL patient cohort *vs*. healthy bone marrow controls. Comparison of *GLUT1*^high^ expression *vs. GLUT1*^low^ expression in **H** overall survival (OS), (**I**) relapse-free survival (RFS) in Ph+ ALL patient cohort. **P < 0.01, ***P < 0.001, ns no significance.

To further understand the underlying mechanism of the synergy, we identified the drug-target genes from the databases of SwissTargetPrediction, SuperPred, SEA Search Server, and PharmMapper databases. Results showed that 822 differentially expressed genes (DEGs) of the drug-target genes were associated with imatinib and ponatinib, and 524 DEGs for CX-4945. Moreover, we analyzed the GSE7186 datasets from a Ph+ ALL cohort and identified a total of 2343 disease-target genes (Supplementary Fig. S13A). Then, we overlapped the two drug-target genes and the disease-target genes, and a total of 65 overlapped DEGs were identified as shown by a Venn diagram plot (Supplementary Fig. S13B) and heatmap (Fig. 5A). PPI analysis indicates the correlation of these 65 DEGs (Supplementary Fig. S13C), and the *GLUT1* is one of the top target gene of glycolysis signal as the primary pathway after the combined therapy.

Next, we investigate the effect of *GLUT1* knockdown and glycolysis in SUP-B15 cells. The *GLUT1* gene was efficiently knocked down in SUP-B15 cells by *GLUT1* shRNA (sh*GLUT1*) in both mRNA (Supplementary Fig. S14A) and protein (Fig. 5B) levels. sh*GLUT1* significantly suppressed the cell proliferation (Fig. 5C), induced G0/G1 phase arrest (Supplementary Fig. S14B), and apoptosis (Fig. 5D, Supplementary Fig. S14C) in the cells. Simultaneously, sh*GLUT1* significantly decreased glucose consumption (Fig. 5E) and lactic acid production (Fig. 5F) compared to shNC. These data indicated that the *GLUT1* knockdown suppresses cell proliferation and induces apoptosis of SUP-B15 cells by suppressing glycolysis.

To determine the clinical significance of *GLUT1* in Ph+ ALL, we further examined the *GLUT1* expression in 20 Ph+ ALL cohorts (Supplementary Table S1) compared to that of 20 normal controls. Results showed that *GLUT1* was significantly overexpressed in Ph+ ALL patients (Fig. 5G). Then, we divided the Ph+ ALL patients into the *GLUT1*^high^ (*n* = 10) and the *GLUT1*^low^ (*n* = 10) groups based on the *GLUT1* mRNA intensity, and the clinical characteristics of the cohorts are shown in Supplementary Table S4. Results showed that *GLUT1*^high^ expression is associated with a significantly higher relapse rate (Supplementary Fig. S14D), a higher % Ik6^+^ (Supplementary Fig. S14E), shorter OS (Fig. 5H, *P* < 0.05), and RFS (Fig. 5I, *P* < 0.05) compared to the *GLUT1*^low^ cohort.

Taken together, these results indicate that *GLUT1* expression is elevated in Ph+ ALL patients, and high *GLUT1* expression is associated with poor outcomes in these patients.

### Combination of imatinib or ponatinib with CX-4945 down-regulates *GLUT1* and suppresses glycolysis in Ph + ALL patient samples

We first examined the effect of combining imatinib or ponatinib with CX-4945 on *GLUT1* expression in SUP-B15 cells. Results showed that the combination significantly suppressed *GLUT1* expression at both the mRNA (Supplementary Fig. S15A) and protein (Supplementary Fig. S15B) levels. Then, we measured the effects of the combination on glucose consumption, lactic acid production, and energy metabolism compared to either single drug alone. Results showed that the combination of imatinib or ponatinib with CX-4945 significantly decreased glucose consumption (Supplementary Fig. S15C) and lactic acid production (Supplementary Fig. S15D) in the cells. Moreover, the Seahorse assay was performed to measure the extracellular acidification rate (ECAR), which reveals the detailed cellular metabolism alterations. Results showed that the combination of imatinib or ponatinib with CX-4945 significantly reduced glycolysis, glycolytic capacity, and glycolytic reserve compared to either single drug (Supplementary Fig. S15E–H).

**Fig. 6:**
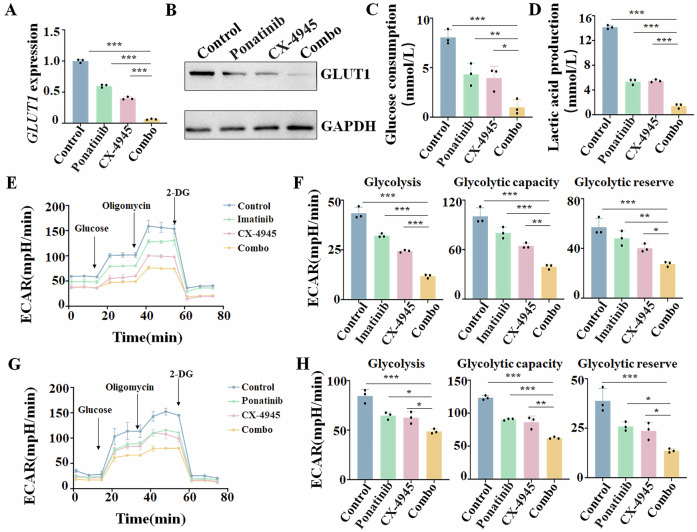
Effect of imatinib or ponatinib with CX-4945 on aerobic glycolysis in primary cells from Ik6^+^ Ph + ALL patient (Pt 2). Expression of *GLUT1* in **A** mRNA level detected by RT-qPCR and **B** protein level detected by Western blot in primary cells from the Ik6^+^ Ph+ ALL patient. Comparison of **C** glucose consumption and **D** lactic acid production in primary cells. **E**–**H** Comparison of the **E**, **G** extracellular acidification rate (ECAR) detected by Seahorse XF96 Extracellular Metabolic Flux Analyzer in primary cells. **F**, **H** Statistical analysis of ECAR to compare the glycolysis, glycolytic capacity, and glycolytic reserve in the four groups. Primary cells were treated with Vehicle control, imatinib (4 μM) or ponatinib (100 nM), CX-4945 (5 μM), and the combination for 48 h. *P < 0.05, **P < 0.01, ***P < 0.001.

We further observed the effect of the combination on *GLUT1* expression and glycolysis in three patient samples (Pt 1-3). CX-4945 significantly inhibited the expression of *GLUT1* at both mRNA (Supplementary Fig. S16A) and protein (Supplementary Fig. S16B) levels in the samples. Combination of imatinib or ponatinib with CX-4945 significantly inhibited the expression of *GLUT1* at mRNA (Fig. 6A, Supplementary Fig. S16C, E, G) and protein (Fig. 6B, Supplementary Fig. S16D, F, H) levels, glucose consumption (Fig. 6C, Supplementary Fig. S17A, C, E), and lactic acid production (Fig. 6D, Supplementary Fig. S17B, D, F) in Pt 1 (Supplementary Figs. S16C, D, S17A, B), Pt 2 (Fig. 6A–D, Supplementary Figs. S16E, F, S17C, D), and Pt 3 (Supplementary Figs. S16G, H, S17E, F) samples. Moreover, the Seahorse assay showed that the combination of imatinib or ponatinib with CX-4945 significantly reduced glycolysis, glycolytic capacity, and glycolytic reserve compared to either single drug in the Pt 2 sample (Fig. 6E–H).

Taken together, these results indicated that the combination of imatinib or ponatinib with CX-4945 not only downregulates the *GLUT1* expression but also suppresses glycolysis in Ph+ ALL cells.

In addition, we examined the effect of sh*GLUT1* on the sensitivity of imatinib or ponatinib and CX-4945, respectively. Results showed that sh*GLUT1* significantly increased the cell proliferation arrest upon imatinib (Supplementary Fig. S18A) or ponatinib (Supplementary Fig. S18B) and CX-4945 (Supplementary Fig. S18C) treatment in SUP-B15 cells compared to that of shNC control cells. The SUP-B15-sh*GLUT1* or SUP-B15-shNC cells were also treated with the combination of various doses of imatinib or ponatinib with 5 μM CX-4945 for 48 h. Results showed that the combination significantly suppressed cell proliferation in SUP-B15-sh*GLUT1* cells compared to SUP-B15-shNC cells (Supplementary Fig. S18D, E). CalcuSyn analysis revealed that the combination had a synergistic effect on cell proliferation arrest in SUP-B15-sh*GLUT1* cells (Supplementary Fig. S19C, D) and SUP-B15-shNC (Supplementary Fig. S19A, B) cells. Then, we compared the difference of the combination-mediated proliferation arrest between sh*GLUT1 vs*. shNC cells, and the results showed that sh*GLUT1* significantly sensitizes the effect of the combination compared to that of shNC control (Supplementary Fig. S18F).

These data indicated the *GLUT1* and glycolysis dependence in the combination-mediated cell proliferation arrest in Ph+ ALL cells.

### IKAROS induces transcriptional repression of *GLUT1* and glycolysis suppression

**Fig. 7:**
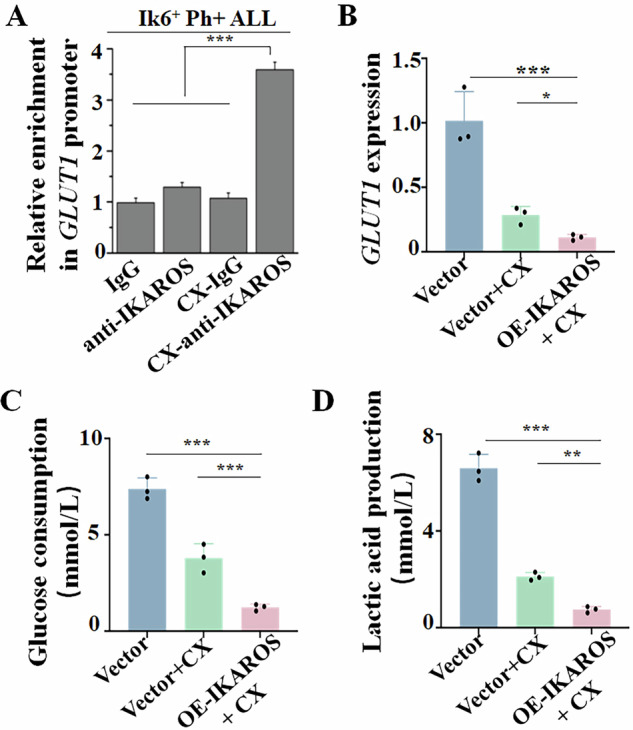
IKAROS represses *GLUT1* transcription and glycolysis in Ph + ALL. **A** Effect of CX-4945 (CX) on IKAROS binding enrichment in the promoter of *GLUT1* gene of Ph+ ALL primary cells with Ik6 by qChIP assay. Cells were treated with 10 μM CX-4945 for 48 h. Comparison of CX on **B** *GLUT1* mRNA level, **C** glucose consumption, and **D** lactic acid production in SUP-B15 cells with IKAROS overexpression (OE-IKAROS + CX) *vs*. Vector-only (Vector+CX) or Vector-only control (Vector), respectively. Cells were treated with 5 μM CX-4945 for 48 h. *P < 0.05, **P < 0.01, ***P < 0.001.

IKAROS is a key leukemia suppressor through transcriptional repression of oncogenes in high-risk B-ALL. As overexpression of IKAROS can potentiate the inhibitory effect of CX-4945 (Fig. 2, Supplementary Fig. S5), we evaluated whether IKAROS was capable of transcriptional repression of *GLUT1*. Our ChIP-seq data showed that IKAROS binding peaks in B-ALL cell lines and patient samples (Supplementary Fig. S20A), and CX-4945 treatment increases the IKAROS binding to the promoter of *GLUT1* genes in B-ALL cells (Supplementary Fig. S20B). qChIP assay confirmed that CX-4945 treatment significantly increases the IKAROS binding in the promoter of the *GLUT1* gene in the Ph+ ALL cells with or without Ik6 (Fig. 7A, Supplementary Fig. S20C, D). IKAROS expression in SUP-B15 cells significantly suppresses the *GLUT1* expression in the mRNA level compared to that of the Vector-only control (Supplementary Fig. S20E). The luciferase reporter assay demonstrated that IKAROS suppresses the promoter activity of the *GLUT1* gene (Supplementary Fig. S20F). Moreover, IKAROS expression significantly sensitizes CX-4945-induced repression of *GLUT1* transcription in SUP-B15 cells (Fig. 7B). These data indicate IKAROS directly represses *GLUT1* transcription.

In addition to the CX-4945-induced suppression of glycolysis in Ph+ ALL cells, we also observed that IKAROS overexpression reduced glucose consumption (Fig. 7C) and lactic acid production (Fig. 7D) in SUP-B15 cells.

Taken together, these results indicated that IKAROS directly represses *GLUT1* transcription and suppresses glycolysis in Ph+ ALL cells. CX-4945 sensitizes the efficacy of imatinib on Ph+ ALL by restoring IKAROS function on transcriptional repression of *GLUT1* and then inducing the suppression of glycolysis in the cells.

Our results reveal a new mechanism to overcome the TKI resistance *via* restoring transcriptional repression of *GLUT1* and further suppression of aerobic glycolysis in Ph+ ALL (Supplementary Fig. S21).

## Discussion

Even with the continuous upgrading of TKIs, half of patients with Ph+ ALL still experience poor treatment effect [56]. Other than TKI resistance responsible for the poor treatment effect, broad-spectrum gene analysis shows that *IKZF1* is a frequently mutated gene in ALL [52]. Consistently, *IKZF1* genetic defects appear in more than 80% of Ph+ ALL. A European study of Ph+ ALL found that patients with *IKZF1* gene mutations had a worse prognosis: 4-year disease-free survival (DFS) of 51.9 ± 8.8% *vs* 78.6 ± 13.9% (*P* = 0.03) compared with WT patients, even when treated with imatinib; the 4-year event-free survival was 20% lower than that of WT patients [57]. We reported that CK2 activity is elevated in high-risk B-ALL, and CK2 inhibitors can restore IKAROS leukemia suppressor function and demonstrate therapeutic efficacy in the disease [40, 41, 43, 49]. Here, we found that IKAROS expression in the Ph+ ALL cells with *IKZF1* deletion increased the sensitivity of TKIs, and CX-4945 sensitized the imatinib or ponatinib therapeutic efficacy in the Ik6^+^ Ph+ ALL-xenograft mouse model. Particularly, the patient for the PDX model had very poor outcome, who died of relapse shortly even when treated with third-generation TKI and blinatumomab. These data together indicate that targeting the CK2/IKAROS axis could sensitize the TKI activity and overcome their resistance in Ph+ ALL.

Moreover, the rapid growth of cancer cells needs large amounts of glucose with an increase in glucose metabolites. The “Warburg effect” can be reflected in a general elevation of metabolic rates to support increased proliferation, as well as a shift in preferred energy source from oxidative phosphorylation to aerobic glycolysis [58]. Increased glucose transporter expression, glucose uptake, and lactate production have been previously reported in *BCR::ABL1*-positive cells, and resistance to TKIs is correlated with sustained glucose metabolism [59, 60]. Imatinib or ponatinib reduced the surface localization of *GLUT1* and further decreased glucose uptake and lactate production. In particular, inhibition of glycolysis can enhance the imatinib cytotoxicity [61]. Consistently, we also observed high expression of *GLUT1* in the B-ALL cohort, and *GLUT1* knockdown significantly suppresses glycolysis along with the cell proliferation arrest, and also facilitates the anti-leukemia effect of imatinib or ponatinib in Ph+ ALL. Our data support the role of *GLUT1* and glycolysis in TKI resistance in Ph+ ALL.

*IKZF1* is known to function as a transcriptional repressor of glucose and energy supply in synergy with other B-lymphoid transcription factors, leading to the enforcement of a state of chronic energy deprivation. Downregulation of *IKZF1* activity generally leads to enhanced malignant characteristics, requiring a higher energy state for proliferation and invasion in ALL cells [62]. Here, we found that CK2 inhibition, restoring IKAROS function on suppression of glycolysis, plays a critical role in sensitizing TKI activity and overcoming TKI resistance. IKAROS directly regulates *GLUT1* transcription; CK2 inhibitor CX-4945 restores IKAROS function and enhances the effect of IKAROS expression on suppression of *GLUT1* transcription and glycolysis in Ph+ ALL. CX-4945 not only sensitizes the TKI anti-leukemia efficacy in vitro and in vivo PDX mouse model but also enhances the suppression of glycolysis in the disease. These data taken together indicated that the IKAROS-mediated suppression of *GLUT1* transcription and aerobic glycolysis is the underlying mechanism of CX-4945 sensitizing the efficacy of TKIs in Ph+ ALL.

Ik6, one of the most common subtypes of IKAROS with impaired function, is present in SUP-B15 cells [63]. In this study, we used the SUP-B15 cells and Ph+ ALL patient samples to dissect the efficacy of targeting the CK2/IKAROS axis on the sensitivity of TKIs to Ph+ ALL, which better reflects the real disease status of Ph+ ALL. Also, these data are further supported by the high expression of *GLUT1* in Ph+ ALL cohorts and its impact on relapse and poor outcomes of Ph+ ALL patients.

Genetic defects are a hallmark of IKAROS dysfunction in B-ALL. We identified that CK2-mediated hyperphosphorylation is another key mechanism leading to the IKAROS dysfunction in B-ALL [40, 41, 43]. CK2 is highly expressed in B-ALL [64]. We also reported that CK2 activity is elevated in the Nalm6 B-ALL cell line and patient samples compared to normal B cell control [40, 41, 43]. CK2 can directly phosphorylate multiple sites on the IKAROS protein, which impairs its function by decreasing DNA-binding ability, promoting degradation, and altering subcellular localization [40, 41, 43, 65]. These sites are primarily located in PEST sequences, rich in proline (P), glutamate (E), serine (S), and threonine (T), in the protein’s N-terminus and C-terminus (e.g., critical phosphorylation sites, amino acids 13 and 294), and CK2 is critical for IKAROS function, particularly during lymphocyte differentiation and as a tumor suppressor [40, 41, 43]. CK2 inhibitor CX-4945 reduces phosphorylation of IKAROS, restoring its DNA binding ability in B-ALL [40, 41, 43]. Our ChIP-seq data in B-ALL cell lines and patient samples showed that CX-4945 increases the IKAROS binding (more binding peaks and higher binding peaks, indicating the increased affinity) in the promoter region of the target genes [40, 41, 43].

Also, *IKZF1* is a codominant expression gene. Almost all *IKZF1* genetic defects (deletion/mutations) are on one allele, which usually encodes a mutated IKAROS protein that loses its DNA binding function; another allele is WT, which can encode the WT IKAROS protein [40, 41, 43]. However, owing to the high expression of CK2 in B-ALL, all the IKAROS proteins are hyperphosphorylated by CK2; IKAROS function is further lost no matter of either *IKZF1-*deletion/mutations or *IKZF1*-WT cells [40, 41, 43]. Therefore, CK2 inhibitor treatment can restore IKAROS function in either *IKZF1* mutant or WT cells, and demonstrates a comparable effect on the PDX mouse model with the *IKZF1* genetic defects compared to that of *IKZF1* WT.

TKI resistance in Ph+ ALL occurs through mechanisms like mutations in the *BCR::ABL1* gene, which are common, and activation of alternative signaling pathways. Despite favorable initial responses to first- or second-generation *BCR::ABL1* TKI–based regimens, relapse resulting from variant-acquired resistance is common [66]. Ponatinib is a third-generation TKI designed to effectively inhibit *BCR::ABL1* with or without any single-mutation variants [67]. Cancer cells can bypass the blocked *BCR::ABL1* pathway by activating other signaling cascades, such as the signaling pathways of PI3K/AKT, Src, RAS/RAF, MDR1, PTEN, etc., and other factors include altered drug transport and the deletion of tumor suppressor genes like *ARF* in chronic myeloid leukemia [68–70]. In addition to *IKZF1*, in B-ALL, the common resistance mechanisms underlying TKI resistance include mutations in the target gene itself, as well as alterations in PAX5, SH2B3, and CRLF2, which can lead to increased signaling through the JAK/STAT pathway [71]. Dysfunction of the IKAROS is a hallmark for high-risk B-ALL, and is associated with the relapse and poor outcome in high-risk B-ALL [40, 41, 43, 52, 72]. Here, we found that IKAROS dysfunction is also one of the key mechanisms for the TKI resistance in Ph+ ALL, and restoring IKAROS function by CK2 inhibitor CX-4945 enhances the sensitivity of TKIs to Ph+ ALL by suppressing *GLUT1* expression and glycolysis, even in the primary cells from the patient who relapsed after the treatment of third-generation TKI and blinatumomab. As IKAROS is one of the major tumor suppressors in B-ALL [40, 41, 43, 52], we consider that aberrant regulation of CK2/ IKAROS on *GLUT1* expression and glycolysis is one of the major mechanisms underlying TKI resistance in Ph+ ALL, although we reported targeting CK2/IKAROS represses the transcription of genes on multi-oncogenic signaling, such as PI3K/AKT, Ras signaling, drug metabolism, CRLF2, SH2B3, etc. [40, 41, 43, 73, 74].

In conclusion, our study demonstrated that targeting the CK2/IKAROS axis by CK2 inhibitor CX-4945 sensitizes the anti-leukemia efficacy of TKIs by suppression of *GLUT1* expression and glycolysis in Ph+ ALL. Our results provide the pre-clinical evidence for this potential combination to prevent resistance and enhance the efficacy of TKIs in Ph+ ALL.

## Supplementary information

Supplemental Information

## Data Availability

The patient datasets for the current study are not publicly accessible by local health research ethics protocols; however, they may be available from the corresponding author.
