# Supplementary material for: Enhancing tyrosine kinase inhibitor sensitivity by restoring IKAROS activity on GLUT1 expression and glycolysis in Philadelphia chromosome-positive acute lymphoblastic leukemia
Source: Leukemia. 2026 Mar 6;40(4):794–805. doi: 10.1038/s41375-026-02898-2 (PMC13056559; doi:10.1038/s41375-026-02898-2)
Supplement: Supplementary file 1 — Supplemental Information [file 41375_2026_2898_MOESM1_ESM.pdf]

Supplemental Information

Enhancing tyrosine kinase inhibitor sensitivity by restoring IKAROS activity on GLUT1 expression and glycolysis in Philadelphia chromosome-positive acute lymphoblastic leukemia

Linyao Zhang[1#], Qi Han[1#], Huimin Xiang[1], Rosa Lapalombella[2], Ann-Kathrin Eisfeld[2], Walter G Hanel[2], Jonathan E Brammer[2], Alice S Mims[2], Jennifer A Woyach[2], Chunhua Song[2*], Zheng Ge[1*]

[1]Department of Hematology, Zhongda Hospital, School of Medicine, Southeast University, Institute of Hematology Southeast University, Nanjing 210009, China

[2]Division of Hematology, The Ohio State University Wexner Medical Center, the James Cancer Hospital, Columbus, OH 43210, USA

#These authors contributed equally to the work

*Correspondence to:

Zheng Ge, M.D., Ph.D.

Department of Hematology, Zhongda Hospital, School of Medicine, Southeast University, Institute of Hematology Southeast University

87 Dingjiaqiao Street, Nanjing 210009, China,

Telephone: +86 25-83262468; Fax: +86 25-83262471

E-mail: zhengge@seu.edu.cn

ORCID: https://orcid.org/0000-0001-8028-1612

OR

Chunhua Song MD, PhD

Division of Hematology, The Ohio State University Wexner Medical Center, The James Comprehensive Cancer Center

536 Biomedical Research Tower, 460 W. 12th Ave., Columbus, OH 43210, USA

Telephone: 614-2928715; Fax: 614-293-7526

E-mail: chunhua.song@osumc.edu

ORCID: https://orcid.org/0000-0002-4081-2543

Supplemental methods and materials

Cell lines and cell culture

B-cell acute lymphoblastic leukemia (B-ALL) cell line SUP-B15, obtained from ATCC, USA, expresses the *BCR::ABL1* gene and its fusion protein P190 and contains the IKAROS isoform 6 (Ik6). The cell line and primary cells were cultured in Roswell Park Memorial Institute 1640 medium (RPMI-1640, Gibco, China). All cell culture media contained 10% fetal bovine serum (FBS, Hyclone, China) and were cultured at 37℃ under a 5% CO2 atmosphere. Logarithmic growth-phase cells were used for the experiment. Every two to three days, the medium was changed.

Main reagents and drugs

Imatinib, ponatinib and CX-4945 were obtained from Selleck (Shanghai, China), dissolved in anhydrous dimethyl sulfoxide (DMSO, Sigma, USA), aliquoted, and stored at -80°C.

Cell proliferation assay

This assay was performed as previously reported[1]. The logarithmic growth phase cells were taken and centrifuged at 1,000 rpm for 5 min, the supernatant was discarded, the cells were resuspended by adding culture medium, and the concentration of the cell suspension was adjusted to 4×10^5/mL. The changes in cell proliferation activity of imatinib, ponatinib, or CX-4945, and the combination of imatinib or ponatinib and CX-4945 were measured by cell counting kit-8 (CCK-8, Dojindo, Japan), respectively. 50 μL growth medium and a 50 μL drug medium were added to each well in a 96-well plate, and a total of 7 serial dilutions of the drug were set up, with 3 replicate wells for each concentration. The plates were incubated at 37°C with 5% CO_2 for 24 hrs, 48 hrs, and 72 hrs, respectively. At the end of the experiment, 10 μL of CCK-8 test solution was added to each well, and incubation was continued for 2-4 hrs. Each well's absorbance was determined using a microplate reader (Bio-Tek, USA) at 450 nm. The 50% inhibitory concentration (IC50) is the concentration of an inhibitor needed to inhibit cells 50% of the time. CalcuSyn software (ComboSyn, USA) was used to analyze the synergistic effect. Using Chou-Talalay's quantitative definition of drug combinations, the combination index (CI) theorem determines the additive effect (CI = 1), synergism (CI < 1), and antagonism (CI > 1)[2].

Flow cytometry apoptosis assay

Cells of the logarithmic growth phase were inoculated in 6-well plates at a density of 4×10^5/mL. After the cells were treated for 48 hrs, the cells were collected, washed with cold phosphate-buffered saline (PBS), and suspended in 100 μL of 1× Annexin V binding buffer. Double staining with 5 μL Annexin V-FITC and 5 μL PI (Becton Dickinson, USA) or 5 μL Annexin V-APC (BD, USA) and 5 μL 7-AAD (Becton Dickinson, USA). 15 min at room temperature and protected from light according to the manufacturer's instructions[1]. Before flow cytometry (Thermo, Waltham, USA) analysis, 400 μL of 1× Annexin V binding buffer was added to each sample. FlowJo version 10.0 software was used for data analysis.

Flow cytometry cell cycle assay

Cells were cultured in the same way as for apoptosis. The cells were then collected, washed, resuspended with 1mL of 70% ice-ethanol solution, and fixed overnight at 4°C. Following centrifugation, ethanol disposal, two PBS washes, and the addition of 0.5 mL of propidium iodide solution (Becton Dickinson, USA) to each tube for 15 min at room temperature[1]. The cells were examined using flow cytometry (Thermo, Waltham, USA). DNA distributions were analyzed using ModfitLT software version 5.0 (Verity Software House, Inc., Topsham, ME, USA).

Real-time quantitative Polymerase Chain Reaction (RT-qPCR) assay

The mRNA expression of relevant genes in various treatment groups was found using Real-time quantitative Polymerase Chain Reaction (RT-qPCR). We first extracted total RNA with TRIzol (TaKaRa, Japan). The same amount of RNA from each sample was used for cDNA synthesis using the PrimeScript^{TM} RT Master Mix (Takara, Japan). The amplification reactions utilizing TB Green Premix Ex Taq (Takara, Japan) were carried out using the StepOne Plus analysis system (Applied Biosystems, USA) following the manufacturer's instructions[3, 4]. The levels of the target mRNAs were normalized using the relative abundance of GAPDH mRNA. The comparative Ct method was used to determine the relative expression levels of the target genes, which were reported as $2^{-\Delta\Delta Ct}$ for cell lines. The list of RT-qPCR primers is shown in Table S3.

Western blot

Cells were cultured in the same way as for apoptosis. After that, they were then collected, washed, and lysed using RIPA Lysis Buffer (Beyotime Biotechnology, China), which contains phenylmethylsulfonyl fluoride (PMSF, Beyotime Biotechnology, China). The supernatant was collected, and 2 μL of the supernatant was taken from each group for protein quantification by the BCA protein assay kit (Vazyme, China). The cell lysates were mixed with 5×SDS loading buffer (Beyotime Biotechnology, China) and heated at 100°C for 10 min, but the *GLUT1* protein was not denatured. The protein samples of each group were subjected to SDS-PAGE gel (Epizyme Biomedical Technology, China) along with a pre-stained protein marker (Thermo, Waltham, USA) and separated by constant voltage electrophoresis. The proteins were then transferred to polyvinylidene difluoride membrane (PVDF, Millipore, USA) by wet transfer method, blocked with 5% nonfat milk (Becton Dickinson, USA) for 1 hour, and placed in the corresponding primary antibodies and incubated overnight at 4°C, and washed 3 times for 10 min each with TBST. The primary antibodies information was as follows: anti-GAPDH (Proteintech, NO. 60004-1-Ig), anti-GLUT1 (ABclonal, NO.A6982), anti-IKAROS (Abcam, NO.ab191394). Then placed in the corresponding secondary antibody (Jackson Immuno Research, USA) and incubated for 1 hour at room temperature. The Invitrogen iBright imaging system and an enhanced chemiluminescence assay kit (ECL, Vazyme, China) were used to image the membranes.

Colony-forming assay

The colony-forming assay was performed according to the instruction manual. Briefly, SUP-B15 cells were cultured in logarithmic growth phase and seeded in 35 mm culture dishes at 2×10^4 cells/mL in Methocult H4100 (Stem Cell Technologies, Canada) supplemented with 10% FBS and 30% desired culture medium. Colonies were scored after 10 to 15 days of culture.

Plasmid construction, lentiviral transduction, and target gene knockdown

Following the manufacturer's instructions, the shRNA oligos were subcloned into the lentiviral shRNA vector (pLV3ltr-ZsGreen-Puro-U6) (Corues Biotechnology, China) to create lentiviral shRNA plasmids for *GLUT1*. The DNA sequence of the shRNA oligos is listed in Table S3. The target gene plasmid (Corues Biotechnology, China) was transfected into 293T cells together with packaging plasmids (psPAX2 and pMD2.G). Following the manufacturer's instructions, transfection was encouraged using Effect Transfection Reagent (Vazyme Biotechnology, China). 48 and 72 hrs after transfection, the viral supernatant was extracted and filtered using a 45 μM pore filter. To improve the transduction efficiency, 40 μL/mL HitransG A (GENECHEM, China) was used, and the resultant lentivirus was transduced into SUP-B15 cells. 48 hrs after transduction, infected cells were treated with 4-8 μg/mL puromycin (Selleck, China) to eliminate the

uninfected cells. The methods for preparing stable overexpression *IKZF1* cell lines are the same as above. *IKZF1* cDNA was subcloned into the lentiviral OE-RNA vector (pLV4ltr-PGK-ZsGreen(2A)Puro-CMV)(Corues Biotechnology, China).

Clinical samples and primary cells

All patients provided informed consent, and the study was approved by the Ethics Committee for Clinical Research of Zhongda Hospital Southeast University, and by the tenets of the Declaration of Helsinki. 20 bone marrow (BM) samples from Ph+ ALL patients and 20 normal control samples of mononuclear cells from healthy volunteers were collected from Zhongda Hospital, Southeast University. Three Ph+ ALL patients who had high leukocyte counts agreed to have their primary cells extracted using leukapheresis. In a sterile environment, mononuclear cells were separated using a lymphocyte separation medium (MP Biomedicals, USA), and erythrocytes were lysed by red blood cell (RBC) lysis buffer (Biosharp, China). As previously mentioned, Trizol was used to extract the total RNA from the samples, and the cDNA was ready for RT-qPCR to detect the target genes. After being cultivated in RPMI-1640 with 10% FBS, the primary cells were employed in the investigations.

The comparative Ct method was used to determine the relative expression levels of the target genes, which were reported as $2^{-\Delta Ct}$ for clinical samples. The characterization of the Ph+ ALL patients is shown in Table S1. The primer sequences for RT-qPCR are listed in Table S3.

Table S2 presents the characteristics of three Ph+ ALL patients with elevated leukocyte counts, which were used for *in vitro* and *in vivo* assays. Patient 1 was a relapsed Ph+ ALL, and Patient 3 is a de novo Ph+ ALL. While Patient 2 is $Ik6^{+}$ Ph+ ALL with 90% blasts and 98% *BCR::ABL1* in BM at newly diagnosis. The second-generation TKI (flumatinib) plus prednisolone was given as induction therapy. Although CR was received, *BCR::ABL1* is positive after the first cycle of induction therapy. Then, blinatumomab+ flumatinib was given. However, the patient relapsed one month later, and the third-generation TKI (olverembatinib) plus hyper-CVAD was given. The patient obtained CR but had a second relapse 6 months later and died [Relapse-free Survival (RFS) 2 months, Overall Survival (OS) 11 months].

Xenotransplantation in mice

Every experimental procedure met with the Regulations for the Administration of Affairs Concerning Experimental Animals of China and was carried out with the approval of the Animal Care Committee of Southeast University.

Male *NOD/ShiLtJGpt-Prkdc*^{em26Cd52}*Il2rg*^{em26Cd22}*/Gpt* (NCG) mice aged 4 weeks old were purchased from GemPharmatech Corporation. For the SUP-B15-xenograft (CDX) mouse model, the mouse sample size (8 mice per group) was determined by G*Power with an F-test of the ANOVA: Fixed effects, omnibus, one-way given effect size F (0.63), α err prob (0.05), power (1-β err prob) (0.80), and number of groups (4). A total of 16 mice for each group were used, 8 mice for the efficacy of the drug on leukemia burden, and 8 mice for survival analysis. Dissolve imatinib in sterilized water and CX-4945 in 25 mM Na_2HPO_4 solution. In the preliminary study, we determined the inoculated cell numbers with days of mouse death and the dose of every single drug. After one week of rearing in a specific pathogen-free facility, mice were injected with 2×10^7 SUP-B15 cells via the tail vein. The engraftment was confirmed by ≥ 5% human leukemia blast cells in the peripheral blood of the mice with flow cytometry analysis, mice (n=16 per group) were randomly divided into four groups and received: Vehicle (Group1); Imatinib via intraperitoneal injection at 25 mg/kg/day, 5 times a week for 4 weeks (Group 2); CX-4945 daily via gavage at 100 mg/kg/day for 4 weeks (Group 3) and combination treatment with imatinib and CX-4945 at the same doses as single drug groups (Group 4). Then, when the vehicle mice met the early removal criteria (ERC, moribundity state, which includes conditions including hemorrhage, loss of consciousness, body weight loss of at least 20%, leg paralysis, and substantially impaired ambulation (inability to get food or water). due to the excessive leukemia burden, 8 mice per group

were euthanized to observe the drug efficacy on leukemia burden. The mice's single-cell suspension of spleen or BM cells was collected, and the RBCs were removed with RBC lysis buffer (Biosharp, China). Mouse CD45 (FITC anti-mouse CD45, eBioscience, Cat:11-0451-81) and human CD19 (APC anti-human CD19, biolgend, Cat:302212) specific antibodies were used to stain the cells for 30 minutes. Flow cytometry (Beckman) was then used to calculate the leukemia burden of the mice's spleen and BM. The remaining 8 mice per group were followed until the mice died or met ERC for survival analysis. The dead mice were counted daily, and the Kaplan-Meier method was used to generate the survival curves and analyze the survival difference.

Ik6$^+$ Ph+ ALL patient-derived xenograft (PDX) mouse model

To get enough cells for the PDX mouse model, the patient's BM samples were amplified once in the female NCG mice. The cellular phenotype and genetic changes in the amplified samples from Patient 2 (Pt 2) were verified to agree with the patient's samples using flow cytometry and next-generation sequencing. For Pt 2, different cell counts (0.5×10^7 to 2×10^7 per mouse) were examined in relation to days to death. We found that injecting 1×10^7 Ph+ ALL cells per mouse was the best dose for the PDX mouse model based on the death days.

16 mice in total were used for each group, 8 of which were used to assess the drug's effectiveness on the leukemia load, and 8 of which were used to analyze survival. As previously mentioned, dissolve imatinib in sterilized water and CX-4945 in 25 mM Na_2HPO_4 solution. Ponatinib was vortexed with 0.5% carboxymethylcellulose sodium (CMC-Na) aqueous solution to form a uniform suspension. The engraftment was verified by ≥ 5% human leukemia blast cells in the peripheral blood of the mice using flow cytometry analysis. The mice (n=16 per group) were randomly assigned to six groups and given the following treatments: Vehicle (Group1); Imatinib via intraperitoneal injection at 25 mg/kg/day, 5 times a week (Group 2); Ponatinib daily via gavage at 10 mg/kg/day (Group 3); CX-4945 daily via gavage at 100 mg/kg/day (Group 4), Combination treatment with imatinib and CX-4945 at the same doses as single drug groups (Group 5); and combination treatment with ponatinib and CX-4945 at the same doses as single drug groups (Group 6). Then, when the vehicle mice satisfied the ERC due to the excessive leukemia burden, 8 mice per group were euthanized to assess the drug's effectiveness on leukemia burden. RBC lysis buffer was used to extract the RBC from the mice's single-cell suspension of spleen or BM cells. The cells were stained for 30 minutes using antibodies specific to mouse CD45 and human CD19. The leukemia burden of the mice's spleen and BM was then determined using flow cytometry. For survival analysis, the remaining 8 mice in each group were monitored until they either passed away or met ERC. Every day, the number of dead mice was counted, and the survival curves and survival difference were analyzed using the Kaplan-Meier method.

Drug targets and therapeutic targets analysis

Drug target genes were obtained from SwissTargetPrediction (http://swisstargetprediction.ch/), SuperPred (https://prediction.charite.de/), SEA Search Server (https://sea.bkslab.org/) and PharmMapper (http://www.lilab-ecust.cn/pharmmapper/submitfile.html/) Databases. Download the Ph+ ALL data for the related dataset GSE7186 from the Gene Expression Omnibus datasets (GEO, https://www.ncbi.nlm.nih.gov/gds/). According to the sample conditions of the GSE7186 dataset, it was divided into the normal control group and the Ph+ ALL patients group. The differential expression analysis of all genes between the healthy group and the Ph+ ALL patients group was analyzed using the Limma R package. Disease target genes of Ph+ ALL were screened with the criteria of $|\log FC| > 1$ and adjusted P-value <0.05. Using the online tool (http://bioinformatics.psb.ugent.be/webtools/Venn/) to draw the Venn diagram plot. The overlapped differential expression genes (DEGs) were identified by intersecting the drug targets and disease targets. The GeneMANIA online database (http://genemania.org/) was used for protein-protein interaction (PPI) network analysis.

Luciferase reporter assay

The promoter regions of *GLUT1* were cloned into the pGL2 vector (Promega, Madison, USA). In 293T cells, the transient luciferase assay was carried out. Luciferase activities were calculated as fold change relative to values obtained from pGL2 vector-only control cells and expressed as a percentage of pcDNA3.1-*IKZF1* transfection-induced luciferase activity versus that of pcDNA3.1 vector. All transfection and reporter assays were performed independently with three replicates[3, 4].

Glucose consumption and lactic acid production assay

Cells were inoculated in 24-well plates at a 4×10^5 cells/mL density. Then, cells were treated for 48 hrs. The culture medium supernatant was obtained for glucose consumption and lactic acid production, which were measured using a detection kit (Jiancheng, China) according to the manufacturer's instructions. The absorbance at 505 nm of glucose consumption was measured using the microplate reader (Bio-Tek, USA). The lactic acid production absorbance at 530 nm. At least three independent assays were performed.

Seahorse assay

The extracellular acidification rate (ECAR) was measured using the XF96 Seahorse Extracellular Flux Analyzer (Seahorse Bioscience, USA) to detect real-time changes. Cells were plated at a density of 4×10^5 cells/mL in 6-well plates and then treated with imatinib alone, ponatinib alone, CX-4945 alone, and imatinib or ponatinib with CX-4945 (Combo) for 48 hrs. The resulting cells were collected for the Seahorse assay. Briefly, the XF96-well cell culture microplate (Agilent, USA) was pre-coated Cell-Tak, and the cells were re-inoculated and cultured. 2 mmol/L glutamine was added to the Seahorse XF RPMI base medium and the pH of the solution was adjusted to 7.4 using NaOH. ECAR was simultaneously measured in basal conditions and after the subsequent addition of 10 mM glucose, 1 μM oligomycin, and 50 mM 2-deoxyglucose (2-DG). All measurements were taken following the manufacturer's instructions and analyzed using Seahorse Wave software.

ChIP-Seq assays

Chromatin immunoprecipitation (ChIP)-Seq assays for IKAROS were performed as previously reported[5, 6]. ChIP assays for IKAROS in 697, JM-1, and REH B-ALL cell lines and B-ALL patient cells[7] were performed using an affinity-purified anti-IKAROS antibody (A303-516A, Bethyl, Montgomery, USA).

ChIP-seq data are accessible on Gene Expression Omnibus with an access number of GSE44218 at the following link:
http://www.ncbi.nlm.nih.gov/geo/query/acc.cgi?acc=GSE44218.

Primary B-ALL ChIP-seq data from patients are available on Gene Expression Omnibus with an access number of GSE58825 at the following link: https://www.ncbi.nlm.nih.gov/geo/query/acc.cgi?acc=GSE58825

Quantitative ChIP-qPCR (qChIP) assay

IKAROS qChIP assays ware performed as described previously[7-9]. The cells were collected and linked on ice for 10 minutes in a cross-linking solution containing 1% formaldehyde to cross-link the proteins with the DNA. Cell lysis buffers are then used to lyse the cells and release chromatin. Chromatin was cut into fragments of 200-1000 bp using an ultrasonic crusher. Antibodies are used to precipitate fragments of DNA bound to IKAROS. DNA was eluted with 50 μL elution buffer (10 mM Tris pH 8.0, 1 mM EDTA, 1%SDS). Heat is then used to de-crosslink, release the DNA, and purify the DNA using a DNA purification kit. Finally, specific primers of the *GLUT1* gene promoter region were used for quantitative analysis by qPCR kit. Primers are shown in Table S3.

Statistical analysis

R 4.1.2 was the software platform used for bioinformatic analysis. Calcusyn software was used to calculate the combination index. The survival curves were created, and the survival difference was examined using the Kaplan-Meier method.

IBM SPSS statistical software v26.0 (SPSS Inc., Chicago, IL, USA) was used to conduct the statistical analysis. Prism program v9.0 (GraphPad program, San Diego, CA, USA) was used to create the data graphics. As stated, independent triplication was used for the experiments. The mean ± standard deviation (SD) was used to show the quantitative data, while percentages and the number of cases were used to describe the qualitative data. Student's t-test was used to compare the measurement data between the two groups, and one-way ANOVA was used to compare data from more than two groups. The chi-squared test or Fisher's precision test was used for the comparison of the categorized data between the two groups. $P < 0.05$ was considered to indicate statistical significance.

References

1. Zi J, Han Q, Gu S, McGrath M, Kane S, Song C, et al. Targeting NAT10 Induces Apoptosis Associated With Enhancing Endoplasmic Reticulum Stress in Acute Myeloid Leukemia Cells. Front Oncol. 2020;10:598107.
2. Chou TC, Talalay P. Quantitative analysis of dose-effect relationships: the combined effects of multiple drugs or enzyme inhibitors. Adv Enzyme Regul. 1984;22:27-55.
3. Ge Z, Song C, Ding Y, Tan BH, Desai D, Sharma A, et al. Dual targeting of MTOR as a novel therapeutic approach for high-risk B-cell acute lymphoblastic leukemia. Leukemia. 2021;35(5):1267-78.
4. Song C, Ge Z, Ding Y, Tan BH, Desai D, Gowda K, et al. IKAROS and CK2 regulate expression of BCL-XL and chemosensitivity in high-risk B-cell acute lymphoblastic leukemia. Blood. 2020;136(13):1520-34.
5. Fujiwara T, O'Geen H, Keles S, Blahnik K, Linnemann AK, Kang YA, et al. Discovering hematopoietic mechanisms through genome-wide analysis of GATA factor chromatin occupancy. Mol Cell. 2009;36(4):667-81.
6. Wang Z, Zang C, Rosenfeld JA, Schones DE, Barski A, Cuddapah S, et al. Combinatorial patterns of histone acetylations and methylations in the human genome. Nat Genet. 2008;40(7):897-903.
7. Song C, Gowda C, Pan X, Ding Y, Tong Y, Tan BH, et al. Targeting casein kinase II restores Ikaros tumor suppressor activity and demonstrates therapeutic efficacy in high-risk leukemia. Blood. 2015;126(15):1813-22.
8. Gurel Z, Ronni T, Ho S, Kuchar J, Payne KJ, Turk CW, et al. Recruitment of ikaros to pericentromeric heterochromatin is regulated by phosphorylation. J Biol Chem. 2008;283(13):8291-300.
9. Song C, Pan X, Ge Z, Gowda C, Ding Y, Li H, et al. Epigenetic regulation of gene expression by Ikaros, HDAC1 and Casein Kinase II in leukemia. Leukemia. 2016;30(6):1436-40.

Supplemental Tables

Table S1. Clinical and laboratory characteristics of Ph+ ALL cohort

Patient ID	Gender	Age (years)	WBC ($\times 10^9$/L)	Hb (g/L)	PLT ($\times 10^9$/L)	BM blasts (%)	PB blasts (%)	Treatment response	Karyotype	Relapse	*GLUT1* expression	Survival
1	Male	54	5	82	27	89	40	CR	45,XY,der(13;14)[10]	Yes	High	Death
2	Male	57	115	135	200	81	69	CR	46,XY[3]/46,XY,t(9;22)[17]	No	High	Death
3	Female	63	211.7	81	173	89	94	PR	46,XX,t(9;22)[5]	Yes	High	Survival
4	Male	47	398.4	114	33	80	93	PR	46,XY,2q+,-8,+?10,-15,15q+,+ph[1]	Yes	Low	Death
5	Female	35	29.4	107	16	93	82	CR	46, XX	No	Low	Survival
6	Male	68	6.7	116	21	59	5	CR	46,XY[4]	Yes	High	Death
7	Male	31	154.3	120	144	71	24	CR	45,XY,ider(9q)t(9;22)(q34;q11),add(20)(q13),-22[8]	Yes	Low	Death
8	Male	17	126	123.5	21	64	95	CR	46, XY	No	Low	Death
9	Male	22	300.0	48	111	79	67	NR	46, XY	Yes	High	Death
10	Male	37	5.52	58	5	94	38	CR	46, XY	Yes	High	Death
11	Female	14	32.25	85	9	94	81	CR	46,XX,t(9;22)(q34;q11)[1]/46,XX[2]	No	Low	Survival
12	Male	51	3.7	122	11	96	72	PR	46, XY	Yes	High	Death
13	Female	27	68.35	56	15	98	90	CR	46, XX	No	Low	Survival
14	Female	46	12.1	92	47	95	50	CR	46, XX	Yes	High	Survival
15	Female	55	29.51	51	269	66	63	CR	45,XX,+X,+5,-7,+8,-9,t(9;22)(q34;q11),-11,-18[1]/46,XX,der(9)[2]/50,XX,+X,+8,+8,-16,+19,-22,+2mar[1]/52,XX,+X,-3,+5,+5,+5,+10,-12,+18,+19,-22,+2mar[1]/46,XX[5]	No	High	Death
16	Male	37	392.3	109	15	96	97	CR	46, XY	No	Low	Survival
17	Male	52	2.09	108	53	14	39	CR	46,XY,t(1;11)(q44;q11),add(4)(p16),del(7)(p11),t(9;22)(q34;q11)[1]/46,XY,der(1)[1]/46,XY,der(1),t(9;22)(q34;q11)[1]/46,XY[1]	No	Low	Survival
18	Male	39	14.37	78	131	91	67	NR	46,XY,t(9;22)(q34;q11)[10]	No	Low	Survival
19	Male	15	17.04	78	27	82	79	CR	47,XY,t(9;22)(q34;q11),+mar[1]/46,XY[2]	Yes	High	Survival
20	Male	40	107.4	109	46	63	47	CR	46,XY,t(9;22)(q34;q11)[6]/45,X,-Y,t(9;22),+10,-15[1]/45,XY,8p+,t(9;22),-16[1]/46,XY[2]	No	Low	Survival

WBC, white blood cell; Hb, hemoglobin; PLT, platelet; BM, bone marrow; PB, peripheral blood; CR, complete remission; PR, partial remission; NR, no remission.

Table S2. Characteristics of Ph+ ALL patients for primary cells

Clinical features	1#	2#	3#
Gender	male	male	female
Age (year)	50	57	62
WBC ($\times10^9$/L)	72.14	229.65	129.05
Hb (g/L)	103	116	87
PLT ($\times10^9$/L)	231	34	104
Disease status	relapse/refractory	newly diagnosis	newly diagnosis
Ik6 / fusion gene	*BCR::ABL1*	Ik6, *BCR::ABL1*	*BCR::ABL1*
Karyotype	45, XY, der(7)t(7;12)(p22;q13), t(9;22)(q34;q11.2), -12[13]/46, XY[7]	46, XY, t(9;22)(q34.1;q11.2)[3]/46, XY[3]	46, XX, t(9;22)(q34; q11.2)[18]/46, idem, t(7;10)(p13;p15), de1(12)(p13p11.2)[2]
Flow cytometric immunotyping	cCD79a+CD34+CD19+CD22+CD10+CD33+CD38+CD11b	cCD79a+CD34+CD19+CD10+CD20+CD22+HLA-DR+CD33+CD13+CD38+	P1:cCD79a+CD34+CD19+CD10+CD22+CD33+CD13+CD38+HLA-DR+; P2:cMPO+CD34+CD117+CD13+CD33+CD38+HLA-DR+CD71+
BM blasts (%)	65.2	90	64.8
Treatment response and outcome	The second-generation TKI (flumatinib) plus CTX and Dex was given as induction therapy. After obtaining CR, flumatinib was given as consolidation and maintenance therapy, and the patient is in the CR status at the latest follow-up.	The second-generation TKI (flumatinib) plus prednisolone was given as induction therapy. Although CR was received, *BCR::ABL1* is positive after the first cycle of induction therapy. Then, blinatumomab + flumatinib was given. However, the patient relapsed one month later, and the third-generation TKI (olverembatinib) plus hyper-CVAD was given. The patient obtained CR but had a second relapse 6 months later and died (RFS 2 months, OS 11 months).	The second-generation TKI (flumatinib) plus Dex was given as induction therapy, and the patient is in the CR status at the latest follow-up.

WBC, white blood cell; Hb, hemoglobin; PLT, platelet; BM, bone marrow; TKI, Tyrosine Kinase inhibitor; CTX, Cyclophosphamide; Dex, Dexamethasone; CR, complete remission; Hyper-CVAD, Cyclophosphamide, Vincristine, Doxorubicin, Dexamethasone; RFS, Relapse-free survival; OS, Overall survival

Table S3. Primer sequences for RT-qPCR, shRNA, and qChIP

	Sequences
GLUT1-F	5’-TTGCAGGCTTCTCCAACTGGAC-3’
GLUT1-R	5’-CAGAACCAGGAGCACAGTGAAG-3’
IKZF1-F	5’-AGAAGCCACACTGGAGAACG-3’
IKZF1-R	5’-ATTTGAAGGGCTTCTCCCCG-3’
GAPDH-F	5’-GTCTCCTCTGACTTCAACAGCG-3’
GAPDH-R	5’-ACCACCCTGTTGCTGTAGCCAA-3’
GLUT1-i1F	gatccGGAATTCAATGCTGATGATtcaagagATCATCAGCATTGAATTCCtttttt
GLUT1-i1R	aattaaaaaaGGAATTCAATGCTGATGATctcttgaATCATCAGCATTGAATTCCg
GLUT1-i2F	gatccgCCAAGAGTGTGCTAAAGAAtcaagagTTCTTTAGCACACTCTTGGtttttt
GLUT1-i2R	aattaaaaaaCCAAGAGTGTGCTAAAGAActcttgaTTCTTTAGCACACTCTTGGcg
GLUT1-promoter-F	5’-CTTGAGCCCAGGAGTTTGAG-3’
GLUT1-promoter-R	5’-GCCGAGGCTGTCTTCTTATG-3’

Table S4. Clinical and laboratory characteristics of Ph+ ALL patients with respect to *GLUT1* expression

	GLUT1 high n=10	*GLUT1* low n=10	*P*
Gender, n. (%)			1.0
Female	3(30)	3(30)	
Male	7(70)	7(70)	
Age(years)			0.07
Median(range)	53(15-68)	36(14-52)	
WBC($\times 10^9$/L)			0.29
Median(range)	15(4-300)	88(2-398)	
WBC>30$\times 10^9$/L, n. (%)			0.18
Yes	3(30)	7(70)	
No	7(70)	3(30)	
Hb(g/L)			0.23
Median(range)	82(48-135)	109(56-124)	
PLT($\times 10^9$/L)			0.24
Median(range)	37(5-269)	27(9-144)	
BM blasts(%)			0.47
Median(range)	86(59-96)	86(14-98)	
PB blasts(%)			0.25
Median(range)	65(5-94)	86(39-90)	
Karyotype			1.0
Normal	5(50)	4(40)	
Abnormal	5(50)	6(60)	
Ik6, n. (%)			0.03
Yes	5(50)	0(0)	
No	5(50)	10(100)	
Treatment response, n. (%)			1.0
CR	7(70)	8(80)	
PR/NR	3(30)	2(20)	
Relapse, n. (%)			0.02
Yes	8(80)	2(20)	
No	2(20)	8(80)	
Survival, n. (%)			0.18
Yes	3(30)	7(70)	
No	7(70)	3(30)	

WBC, white blood cell; Hb, hemoglobin; PLT, platelet; BM, bone marrow; PB, peripheral blood; CR, complete remission; PR, partial remission; NR, no remission.

Supplemental Figures

Figure. S1

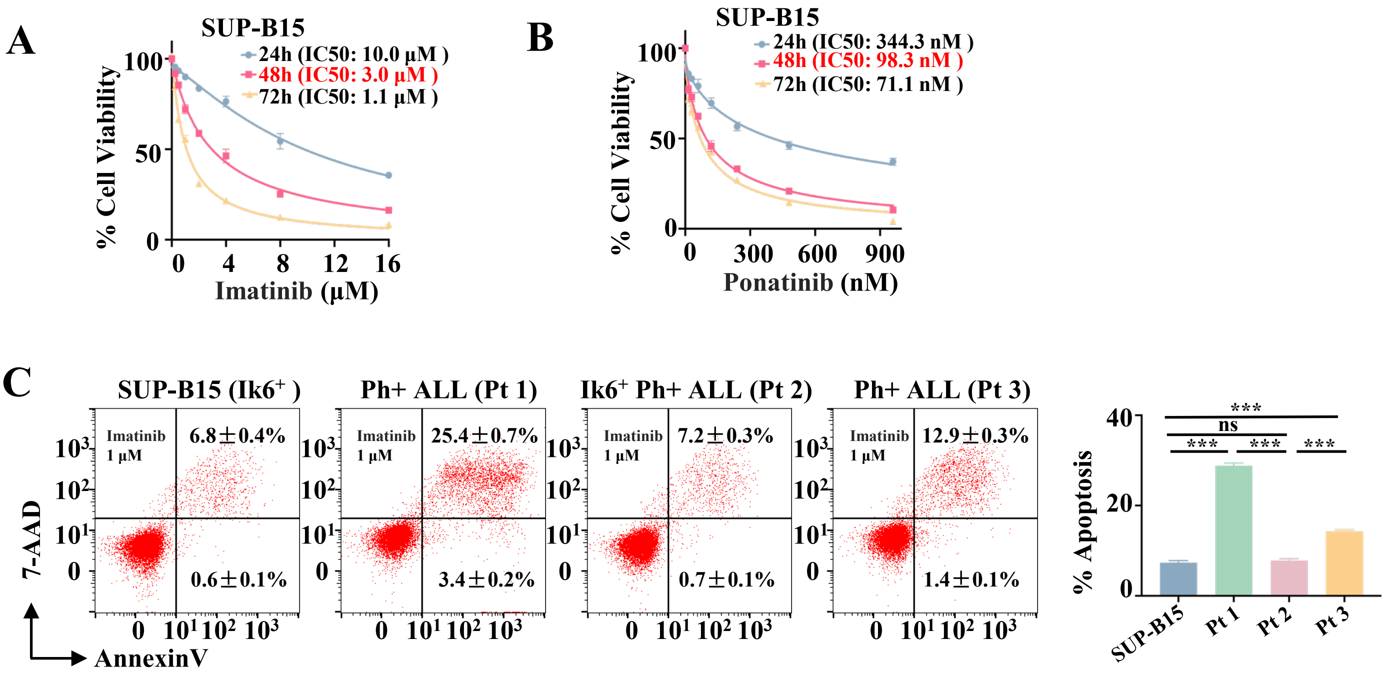

Fig. S1 Comparison of imatinib or ponatinib sensitivity on cell proliferation arrest in SUP-B15 and primary cells. (A-B) Effect of (A) imatinib and (B) ponatinib on cell proliferation of SUP-B15 cells. For A-B, the SUP-B15 cells were treated with various doses of imatinib or ponatinib for 24 hrs, 48 hrs, and 72 hrs, respectively. (C) Effect of imatinib on apoptosis of SUP-B15 and primary cells. The cells were treated with Pt 1's IC50 doses of imatinib (1 μM) for 48 hrs. *** *P* < .001, ns: no significance.

Figure. S2

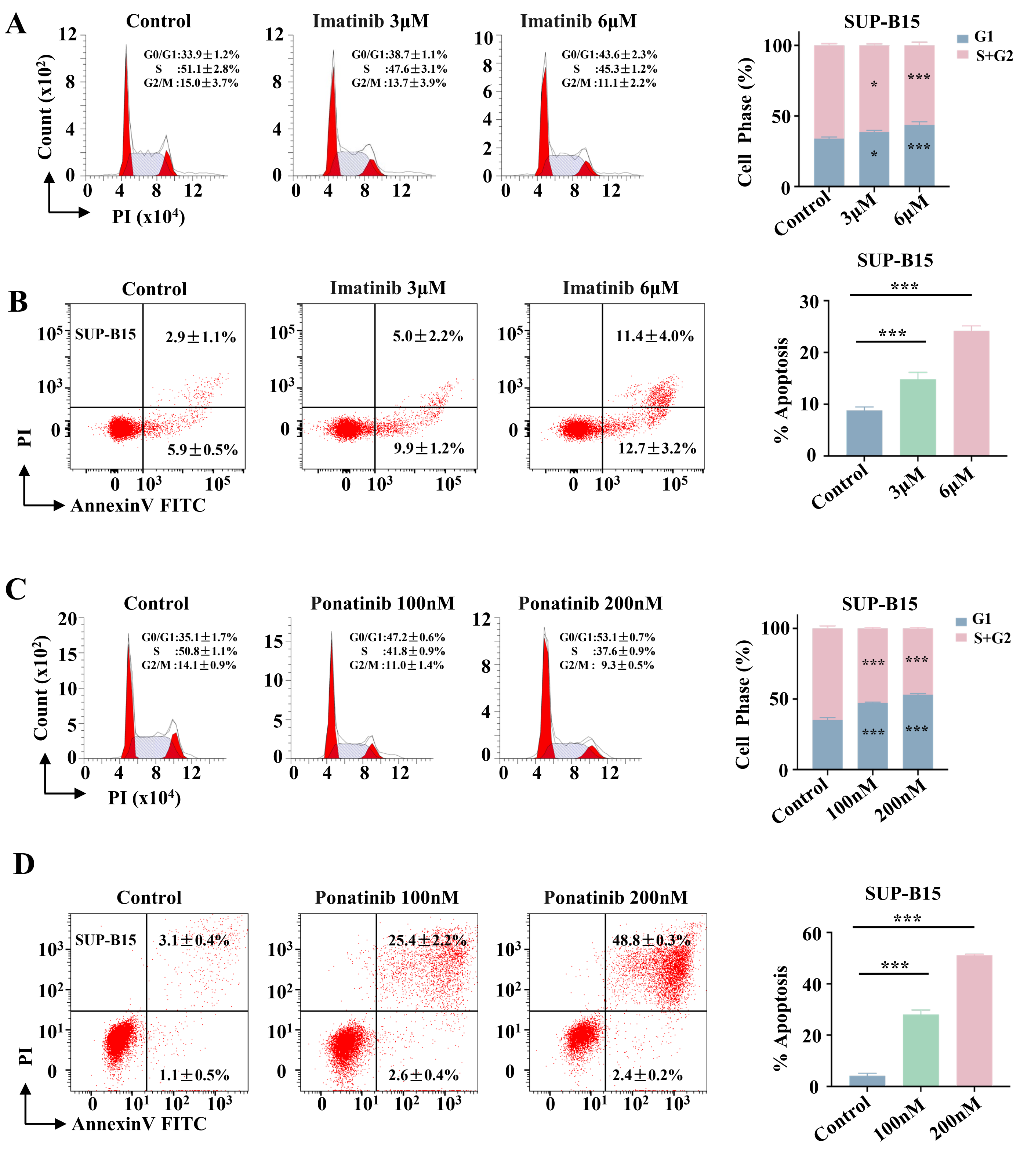

Fig. S2 Effect of imatinib or ponatinib on the SUP-B15 cells. (A-B) Effect of imatinib on (A) cell cycle, and (B) apoptosis in SUP-B15 cells. (C-D) Effect of ponatinib on (C) cell cycle, and (D) apoptosis in SUP-B15 cells. Cells were treated with indicated doses of imatinib or ponatinib for 48 hrs for cell cycle and apoptosis assay. * *P*<.05, *** *P*<.001.

Figure. S3

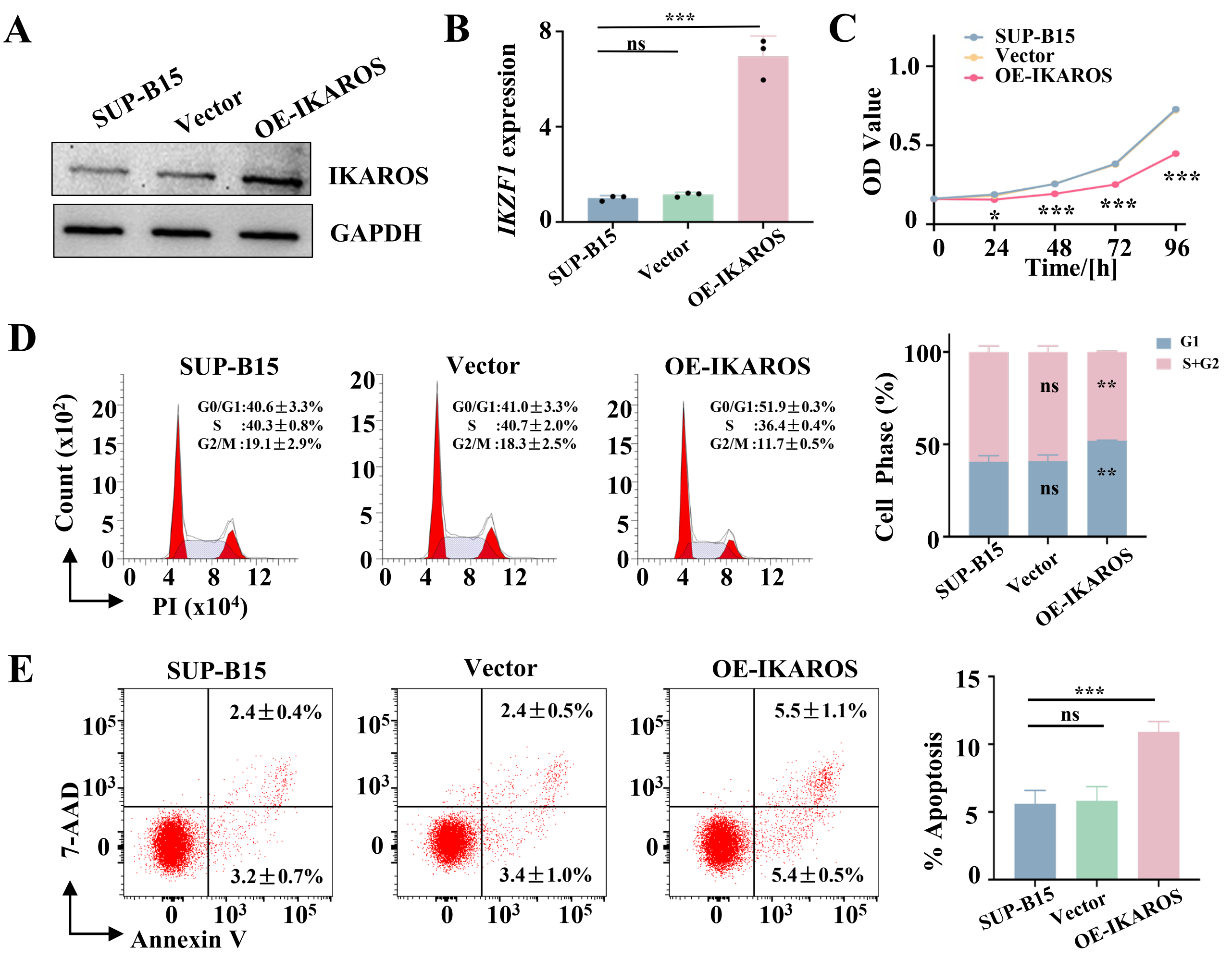

Fig. S3 Restoring *IKZF1* function results in an anti-leukemia effect in SUP-B15 cells. (A) Protein level of IKAROS in SUP-B15 cells overexpressed IKAROS (OE-IKAROS), Vector-only control (Vector), and SUP-B15 cell control (SUP-B15). The cells were transduced with lentiviral IKAROS or Vector, the cell lysate was prepared, and IKAROS was detected by Western blot. (B) The mRNA level of *IKZF1* in SUP-B15 cells with OE-IKAROS, Vector-only control, and SUP-B15 control, which were detected by RT-qPCR. (C-E) Comparison of (C) cell viability, (D) cell cycle progress, and (E) apoptosis in the SUP-B15 cells with OE-IKAROS, Vector-only control, and SUP-B15 control. * $P < .05$, ** $P < .01$, *** $P < .001$, ns: no significance.

Figure. S4

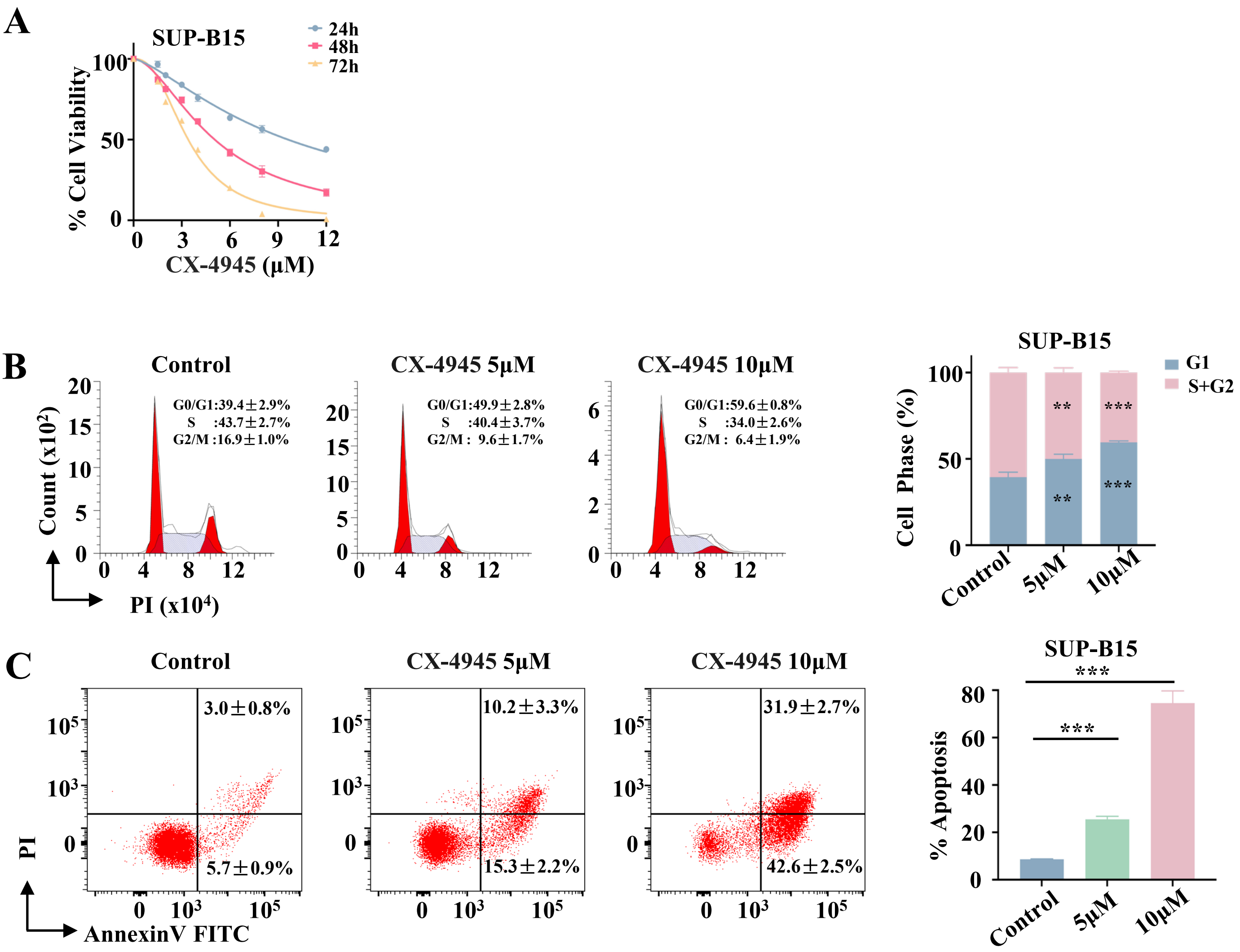

Fig. S4 Effect of CX-4945 on the SUP-B15 cells. (A-C) Effect of CX-4945 on (A) cell viability, (B) cell cycle, and (C) apoptosis in SUP-B15 cells. Cells were treated with indicated doses of CX-4945 for 24 hrs, 48 hrs, and 72 hrs for cell viability assay and 48 hrs for cell cycle and apoptosis assay. ** $P < .01$, *** $P<.001$.

Figure. S5

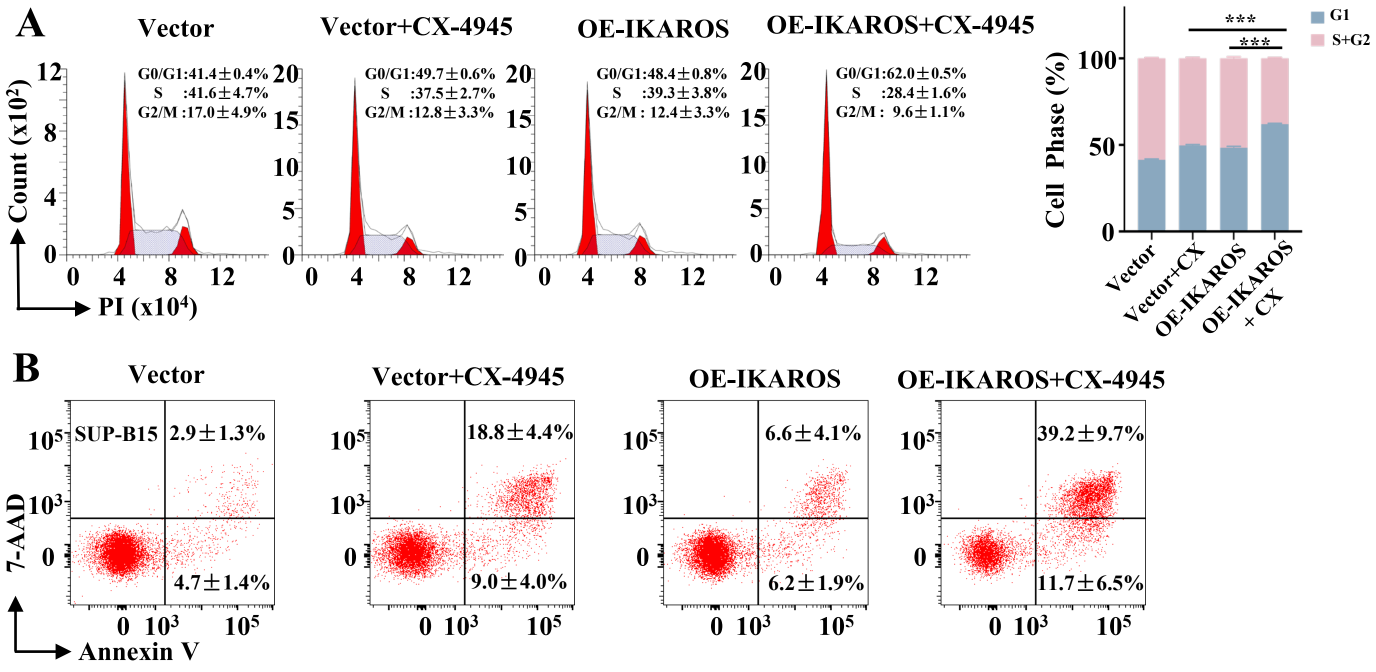

Fig. S5 CX-4945 restores IKAROS function, resulting in an anti-leukemia effect in SUP-B15 cells. (A-B) Comparing the effect of CX-4945 *vs.* Vehicle control on the (A) cell cycle, and (B) apoptosis in SUP-B15 cells with OE-IKAROS *vs.* Vector-only, i.e. in four groups, Vector+Vehicle (Vector), Vector+CX-4945, OE-IKAROS+Vehicle (OE-IKAROS), OE-IKAROS+CX-4945. For A-B, the cells were treated with CX-4945 or Vehicle for 48 hrs. *** *P* < .001.

Figure. S6

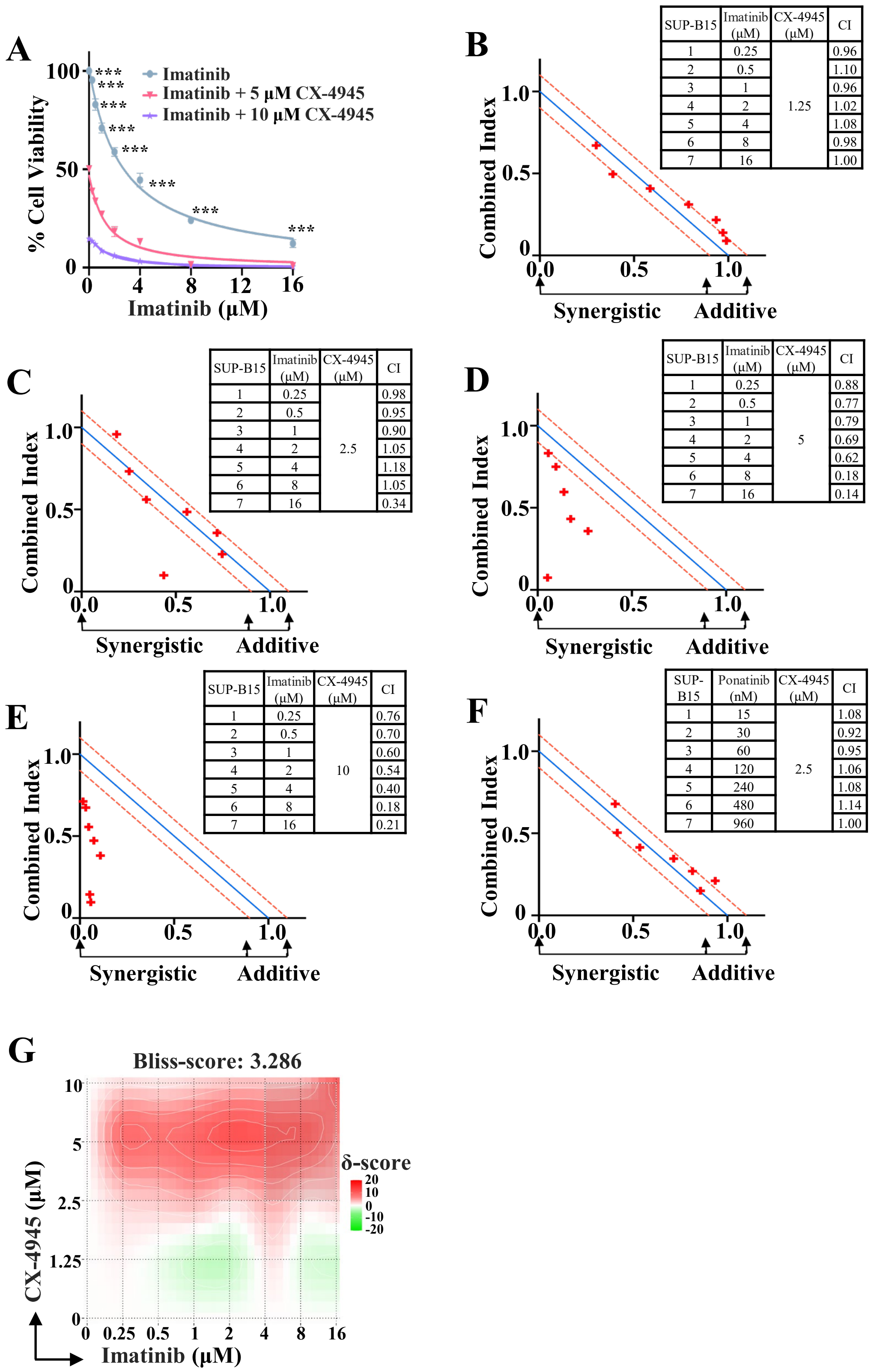

Fig. S6 The synergistic effect of imatinib or ponatinib with CX-4945 in SUP-B15 cells. (A) Effect of the combination of imatinib with CX-4945 on the cell proliferation arrest of SUP-B15 cells. The cells were treated with various doses of imatinib with IC50 and double IC50 CX-4945 for 48 hrs. (B-F) CalcuSyn analysis of the combination of different doses of imatinib or ponatinib with various doses of CX-4945 on cell proliferation arrest in SUP-B15 cells. (G) Synergistic analysis of imatinib with CX-4945 on proliferation arrest with the Bliss models. *** $P < .001$.

Figure. S7

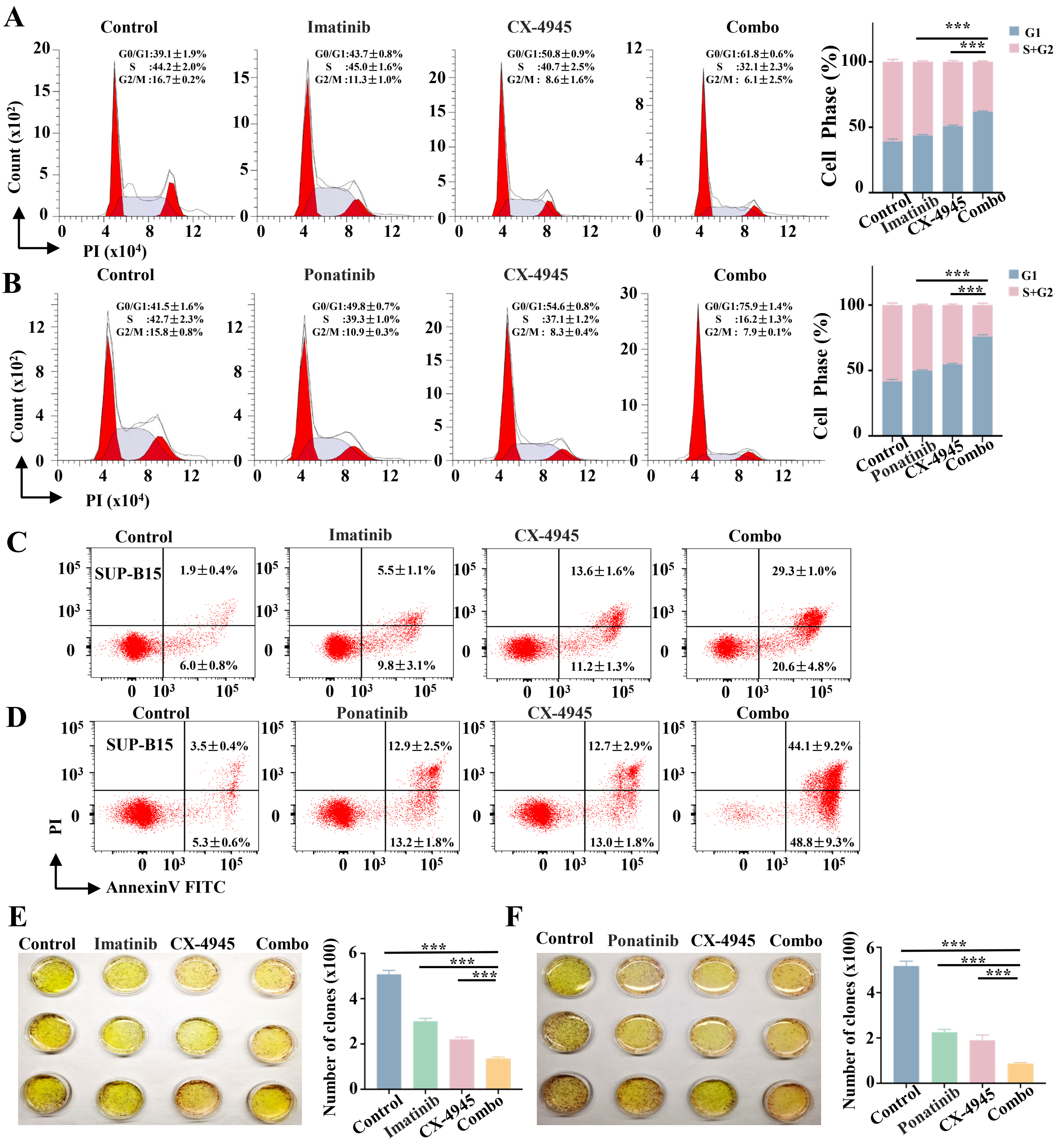

Fig. S7 The synergistic effect of imatinib or ponatinib with CX-4945 in SUP-B15 cells. (A-D) Effect of (A, C) imatinib or (B, D) ponatinib with CX-4945 on (A, B) cell cycle arrest and (C, D) apoptosis in SUP-B15 cells compared to single drug control and vehicle only control. (E-F) Effect of colony-forming in SUP-B15 cells. For A-F, the cells were treated with 3 μM imatinib or 100 nM ponatinib, 5 μM CX-4945, and the combination for 48 hrs. *** $P < .001$.

Figure. S8

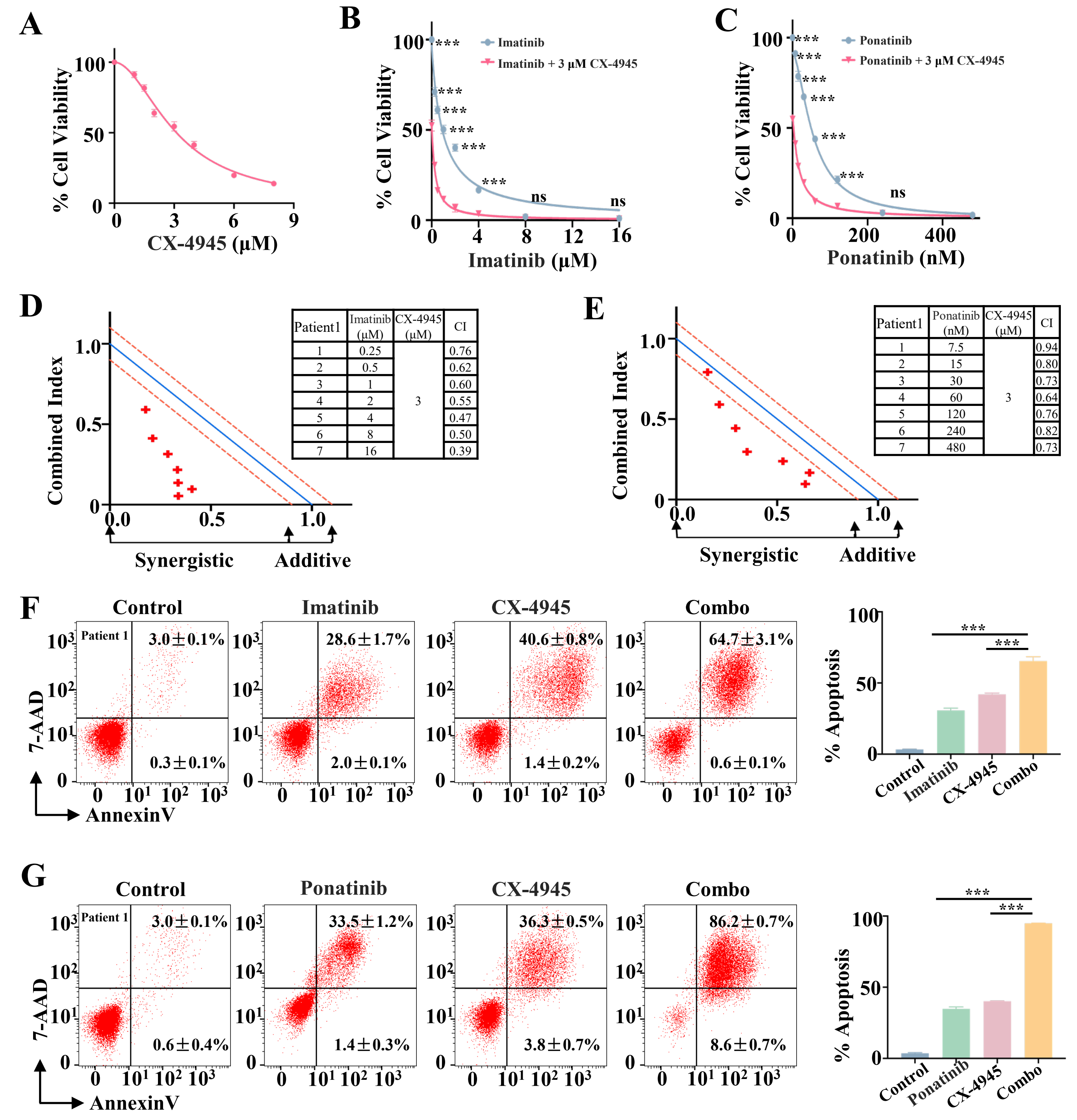

Patient1	Imatinib (μM)	CX-4945 (μM)	CI
1	0.25		0.76
2	0.5		0.62
3	1		0.60
4	2	3	0.55
5	4		0.47
6	8		0.50
7	16		0.39

Patient1	Ponatinib (nM)	CX-4945 (μM)	CI
1	7.5		0.94
2	15		0.80
3	30		0.73
4	60	3	0.64
5	120		0.76
6	240		0.82
7	480		0.73

Fig. S8 The synergistic effect of imatinib or ponatinib with CX-4945 in primary cells from the Ph+ ALL patient (Pt 1). (A) Effect of CX-4945 on cell viability in Ph+ ALL primary cells from Pt 1. Cells were treated with indicated doses of CX-4945 for 48 hrs for the cell viability assay. (B-C) Effect of the combination of (B) imatinib or (C) ponatinib with CX-4945 on the cell proliferation arrest of Pt 1's primary cell. The cells were treated with various doses of imatinib or ponatinib at IC50 concentrations of CX-4945 for 48 hrs. (D-E) CalcuSyn analysis of the combination of proliferation arrest in primary cells. (F-G) Effect of (F) imatinib or (G) ponatinib with CX-4945 on apoptosis in primary cells compared to single drug control and vehicle only control. For F-G, the cells were treated with vehicle control, 1 μM imatinib, 50 nM ponatinib, 3 μM CX-4945, and the combination for 48 hrs. *** *P* < .001, ns: no significance.

Figure. S9

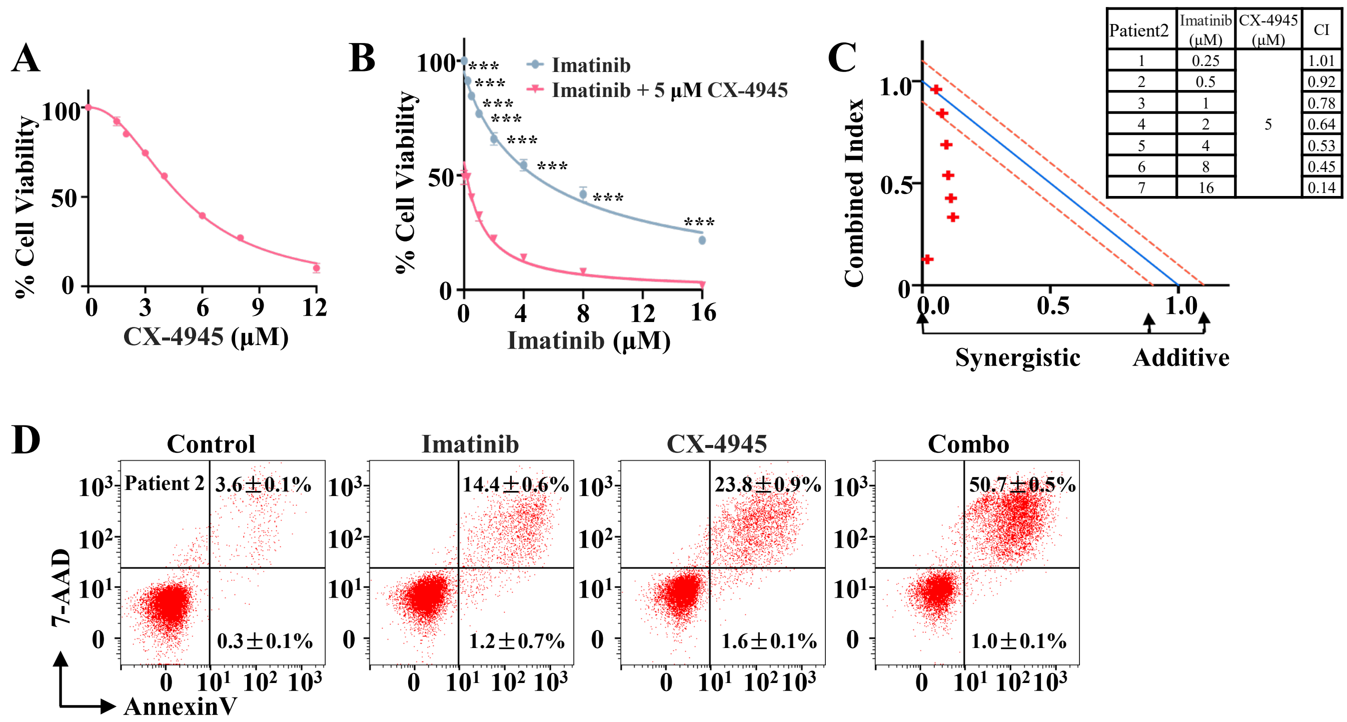

Fig. S9 The synergistic effect of imatinib or ponatinib with CX-4945 in primary cells from the Ik6^{+} Ph+ ALL patient (Pt 2). (A) Effect of CX-4945 on cell viability in Pt 2's primary cells. Cells were treated with indicated doses of CX-4945 for 48 hrs for the cell viability assay. (B) Effect of the combination of imatinib with CX-4945 on the cell proliferation arrest of Pt 2's primary cell. The cells were treated with various doses of imatinib with IC50 CX-4945 for 48 hrs. (C) CalcuSyn analysis of the combination of proliferation arrest in primary cells. (D) Effect of imatinib with CX-4945 on apoptosis in primary cells compared to single drug control and vehicle only control. The cells were treated with vehicle control, 4 μM imatinib, 5 μM CX-4945, and the combination for 48 hours. *** $P < .001$.

Figure. S10

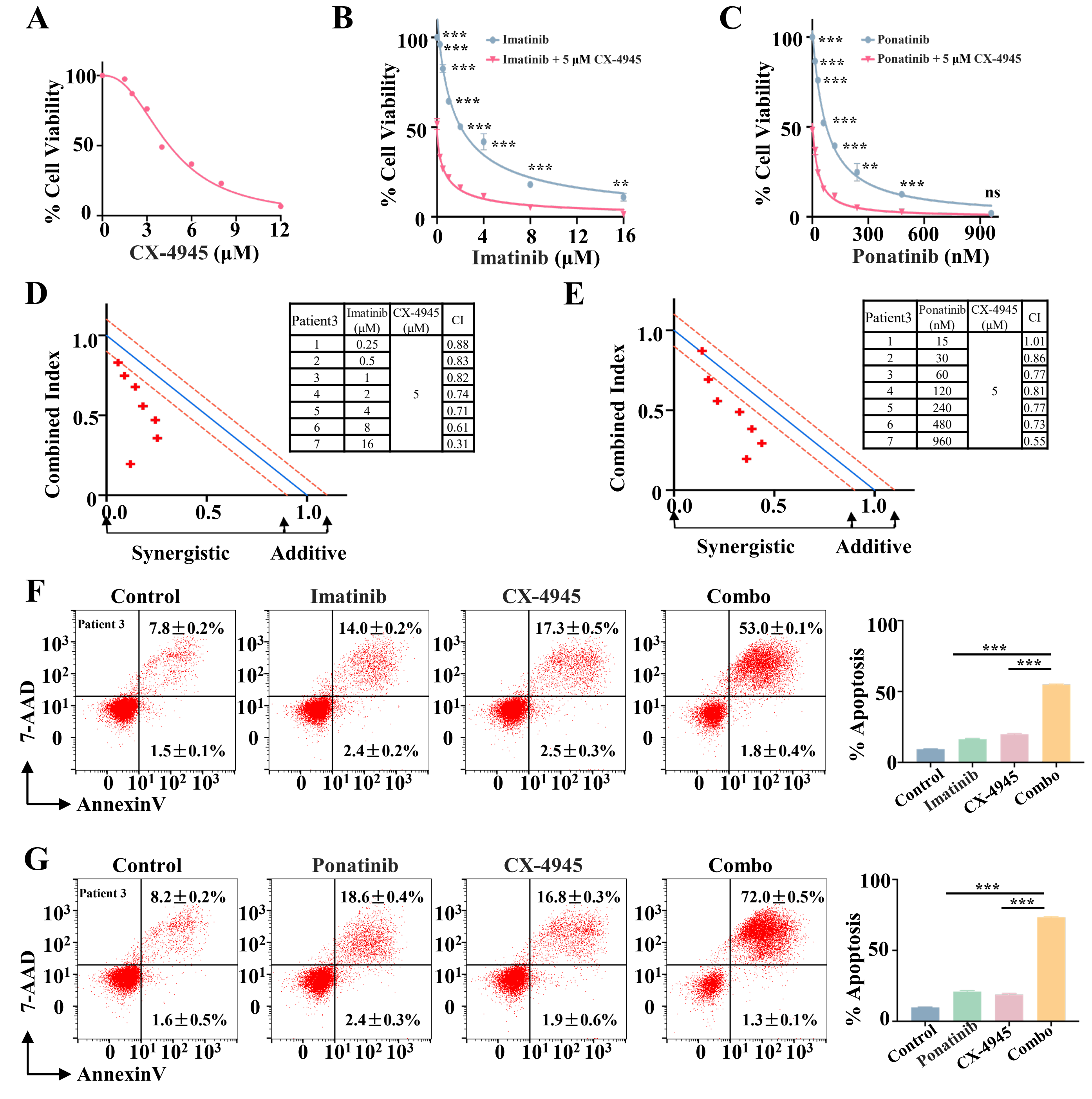

Patient3	Imatinib (μM)	CX-4945 (μM)	CI
1	0.25		0.88
2	0.5		0.83
3	1		0.82
4	2	5	0.74
5	4		0.71
6	8		0.61
7	16		0.31

Patient3	Ponatinib (nM)	CX-4945 (μM)	CI
1	15		1.01
2	30		0.86
3	60		0.77
4	120	5	0.81
5	240		0.77
6	480		0.73
7	960		0.55

Fig. S10 The synergistic effect of imatinib or ponatinib with CX-4945 in primary cells from the Ph+ ALL patient (Pt 3). (A) Effect of CX-4945 on cell viability in Ph+ ALL primary cells from Pt 3. Cells were treated with indicated doses of CX-4945 for 48 hrs for the cell viability assay. (B-C) Effect of the combination of (B) imatinib or (C) ponatinib with CX-4945 on the cell proliferation arrest of Pt 3's primary cell. The cells were treated with various doses of imatinib or ponatinib at IC50 concentrations of CX-4945 for 48 hrs.. (D-E) CalcuSyn analysis of the combination of proliferation arrest in primary cells. (F-G) Effect of (F) imatinib or (G) ponatinib with CX-4945 on apoptosis in primary cells compared to single drug control and vehicle only control. For F-G, the cells were treated with 2 μM imatinib or 80 nM ponatinib, 5 μM CX-4945, and the combination for 48 hrs. ** *P* < .01, *** *P*<.001, ns: no significance.

Figure. S11

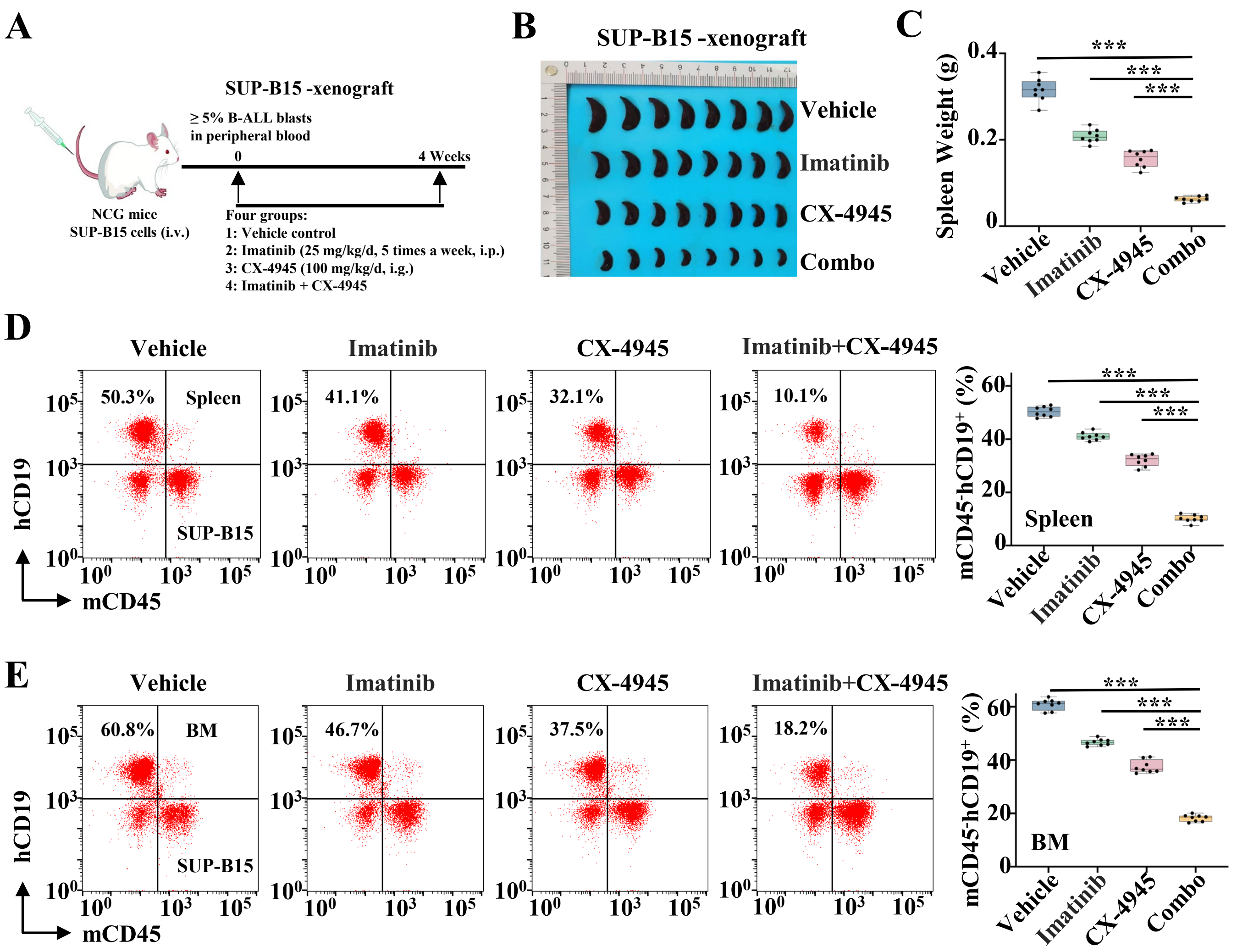

Fig. S11 Synergistic efficacy of imatinib with CX-4945 on leukemia development in the SUP-B15-xenograft (CDX) mouse model. (A) Schematic representation of the human Ph+ ALL SUP-B15 cell-xenograft mouse model (SUP-B15-xenograft) with treatment schemes. SUP-B15 (2×10^7 cells/mouse) cells were intravenously injected into NCG mice, the mice were treated in the four groups for 28 days: group 1 (Vehicle); group 2 (Imatinib 25 mg/kg/day for weekday, i.p.); group 3 (CX-4945 100 mg/kg/day, gavage); group 4 (combo with the same doses as single drug groups). (B-C) Comparison of (B) spleen size and (C) spleen weight of the four groups in the CDX mouse model. (D-E) The representative flow scatter plot and quantitative data of $mCD45^{-}hCD19^{+}$ cells in the (D) spleen and (E) bone marrow (BM) of the CDX mouse model. For D-E, the mice were euthanized after the treatment scheme was completed, the spleens were isolated, and the splenocytes and BM cells were prepared. *** *P*<.001.

Figure. S12

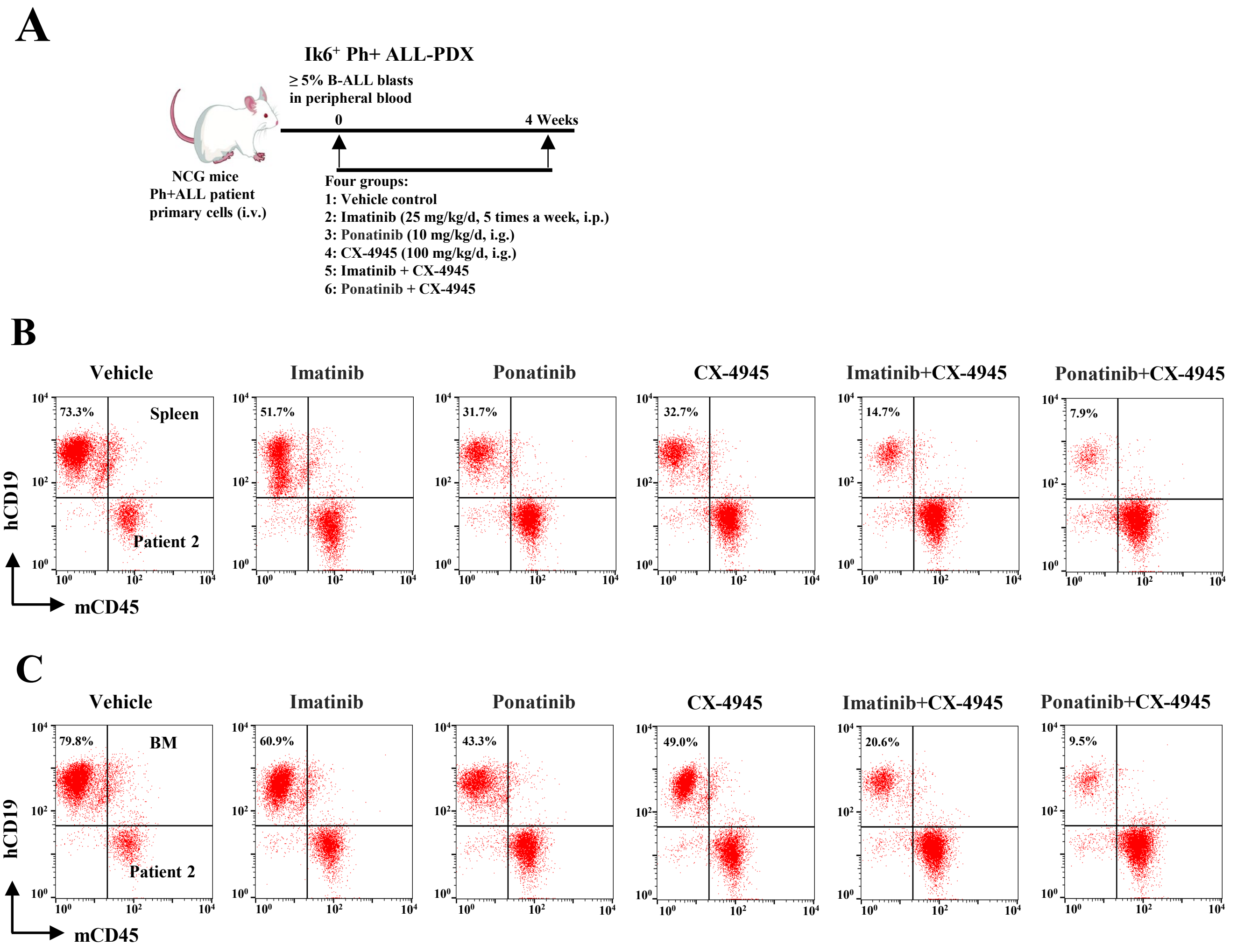

Fig. S12 Synergistic efficacy of imatinib with CX-4945 on leukemia development in Ik6^{+} Ph+ ALL patient-derived xenograft (PDX) mouse model. (A) Schematic representation of the treatment schemes for the PDX mouse model. Primary cells (1×10^{7} cells/mouse) were intravenously injected into NCG mice, the mice were treated in the six groups for 28 days: group 1 (Vehicle); group 2 (Imatinib 25 mg/kg/day for weekday, i.p.); group 3 (Ponatinib 10 mg/kg/day, gavage.); group 4 (CX-4945 100 mg/kg/day, gavage); group 5 (combination of imatinib and CX-4945 with the same doses as single drug groups); group 6 (combination of ponatinib and CX-4945 with the same doses as single drug groups). (B-C) The representative flow scatter plot of mCD45^{-}hCD19^{+} cells in (B) the spleen and (C) bone marrow (BM) of the mice. For B-C, the mice were euthanized after the treatment scheme was completed, the spleens were isolated, and the splenocytes and BM cells were prepared.

Figure. S13

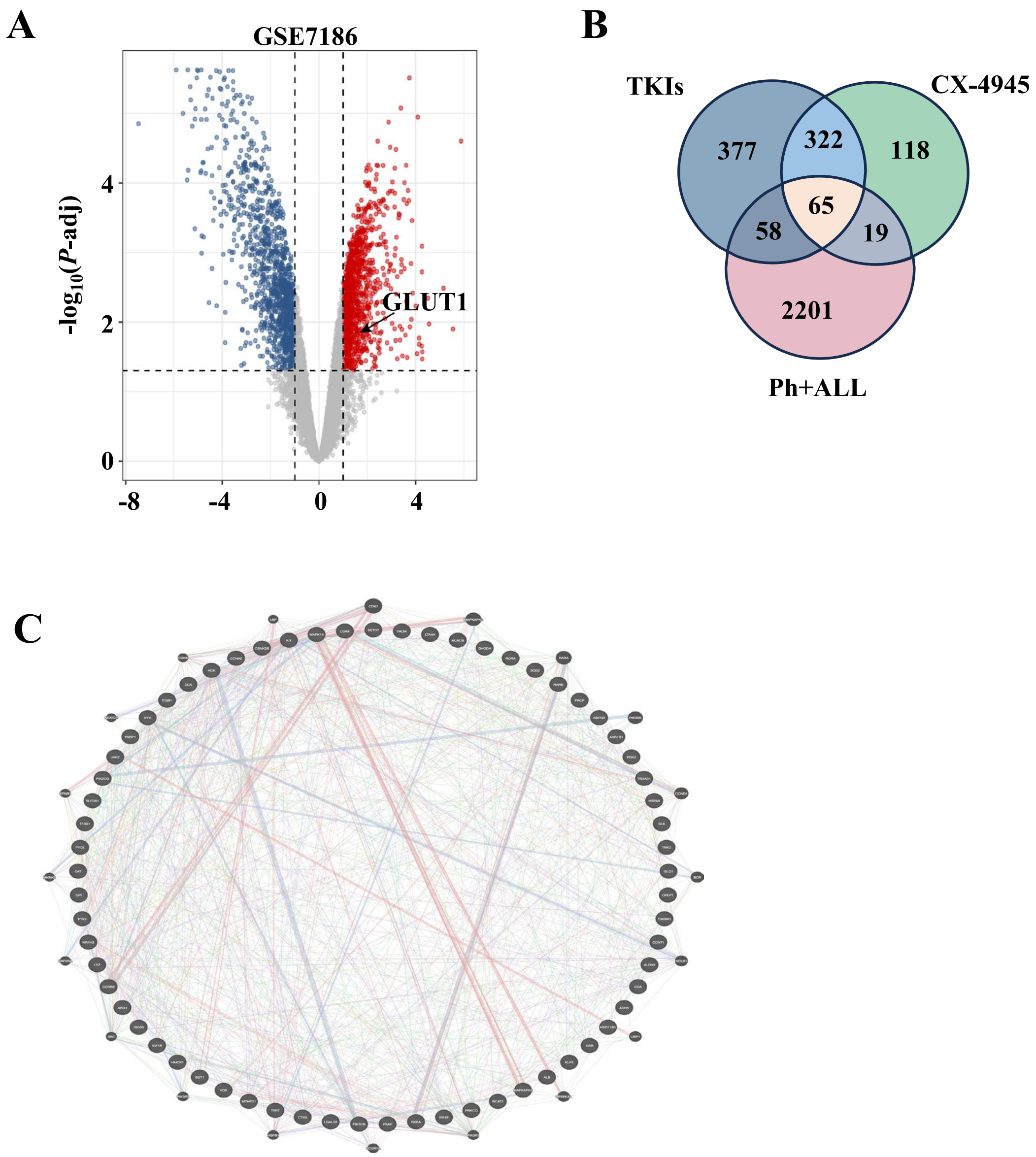

Fig. S13 DEGs of disease-target genes. (A) Volcano plot showing the DEGs in the Ph+ ALL cohort *vs.* normal controls from the GSE7186 database. (B) The Venn plot showing the overlapped DEGs of drug-target genes and disease-target genes. (C) Analysis of the protein-protein interaction network for DEGs with the GeneMANIA database tool.

Figure. S14

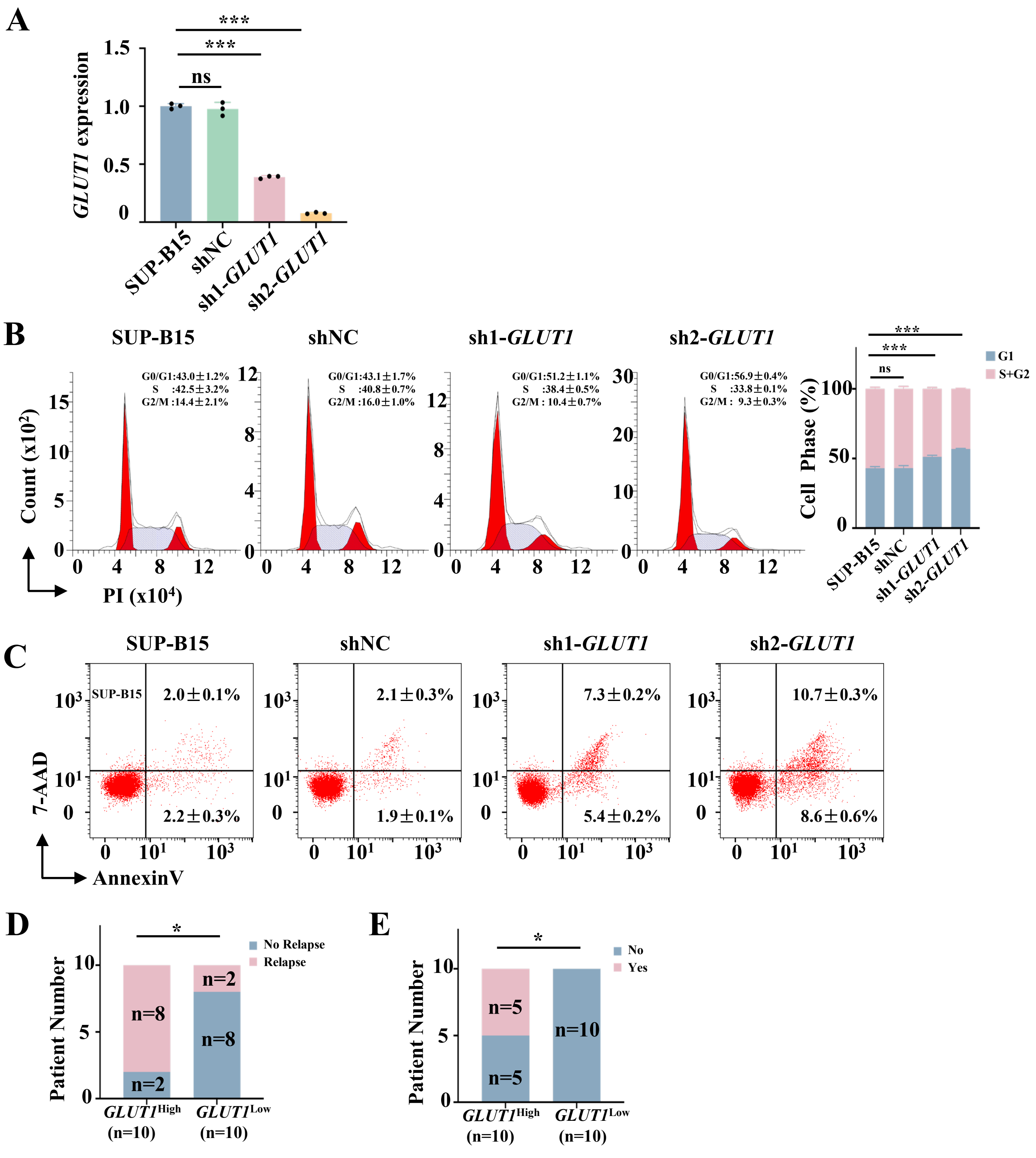

Fig. S14 Roles of *GLUT1* in SUP-B15 cells and Ph+ ALL. (A) Comparison of the *GLUT1* mRNA level in SUP-B15 cells with sh*GLUT1* *vs.* shNC or SUP-B15, detected by RT-qPCR. SUP-B15 cells were stably transduced with lentiviral *GLUT1* shRNA (sh*GLUT1*) or scramble shRNA (shNC). (B-C) Comparison of (B) cell cycle and (C) apoptosis in SUP-B15 cells with sh*GLUT1* *vs.* shNC or SUP-B15. (D-E) Comparison of *GLUT1*^{high} expression *vs.* *GLUT1*^{low} expression in (D) % relapse rate, (E) % Ik6 in Ph+ ALL patient cohort. * $P < .05$, *** $P < .001$, ns: no significance.

Figure. S15

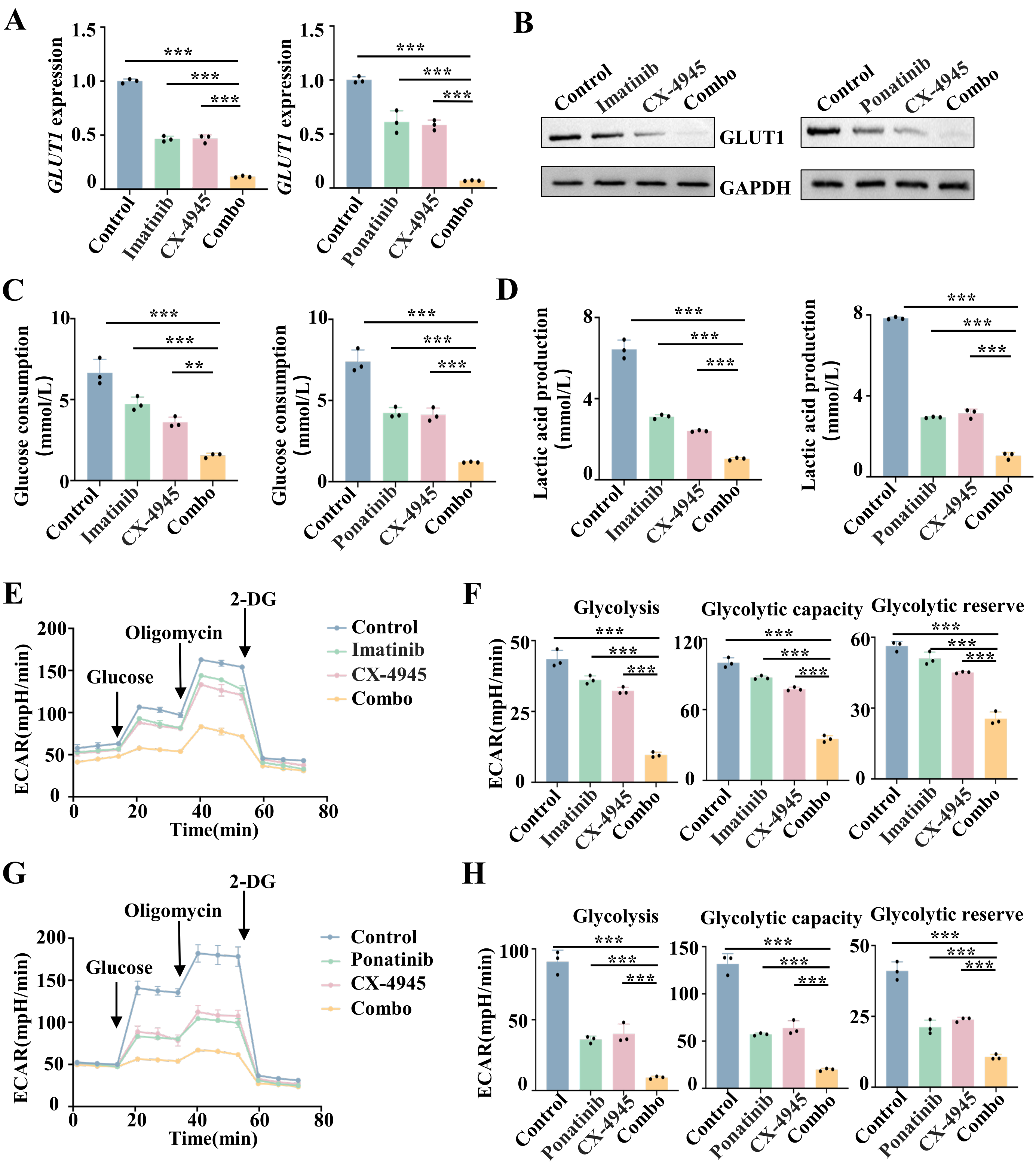

Fig. S15 Effect of imatinib or ponatinib with CX-4945 on aerobic glycolysis in SUP-B15 cells. (A-B) Expression of *GLUT1* in (A) mRNA level detected by RT-qPCR and (B) protein level detected by Western blot in SUP-B15 cells. (C-D) Comparison of (C) glucose consumption and (D) lactic acid production in SUP-B15 cells. (E-H) Comparison of the (E, G) extracellular acidification rate (ECAR) detected by Seahorse XF96 Extracellular Metabolic Flux Analyzer in SUP-B15 cells. (F, H) Statistical analysis of ECAR to compare the glycolysis, glycolytic capacity, and glycolytic reserve in the four groups. SUP-B15 cells were treated with vehicle control, imatinib (3 μM) or ponatinib (100 nM), CX-4945 (5 μM), and the combination for 48 hrs. ** $P < .01$, *** $P < .001$.

Figure. S16

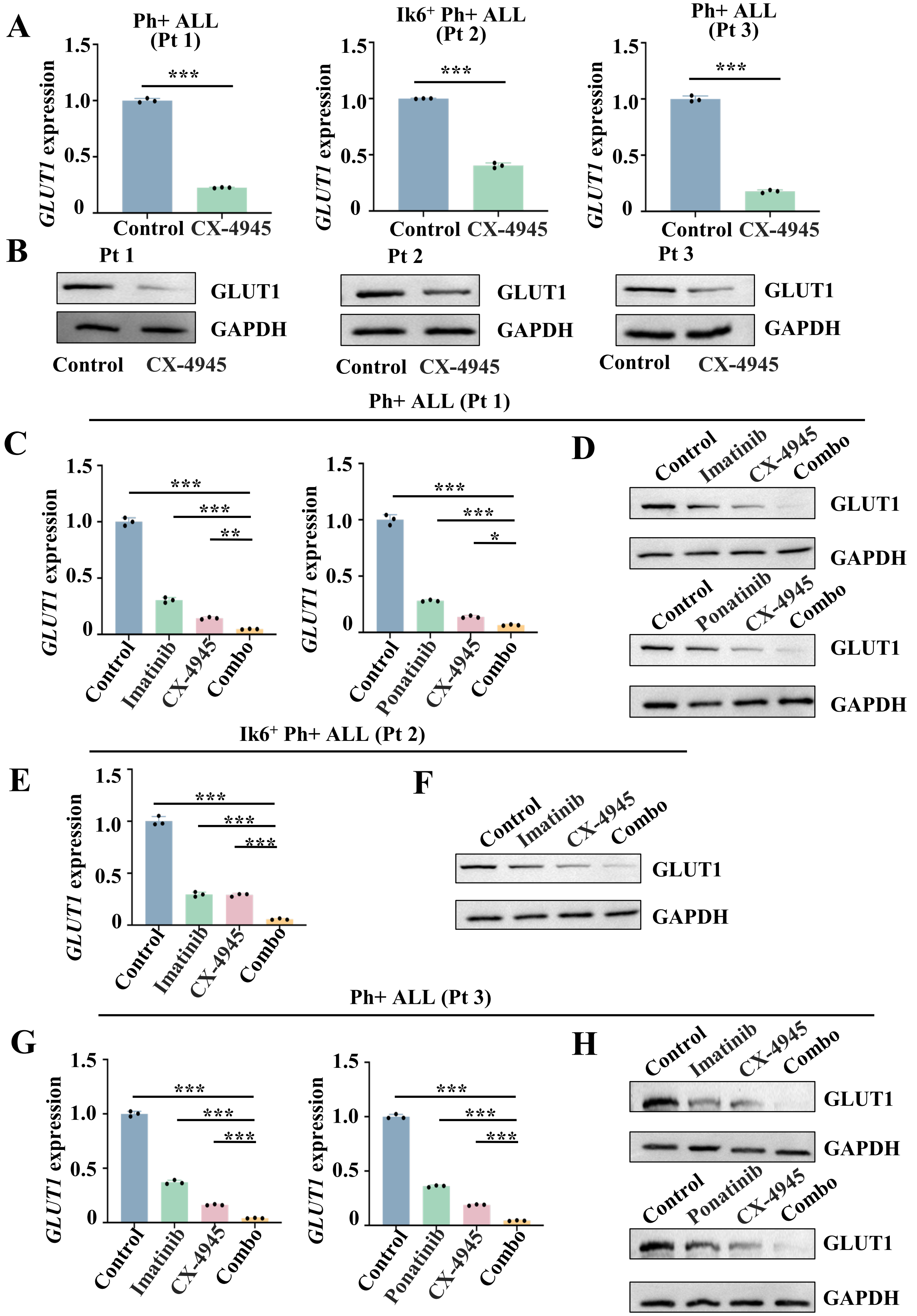

Fig. S16 Effect of imatinib or ponatinib with CX-4945 on *GLUT1* expression in primary cells. (A-B) Expression of *GLUT1* in (A) mRNA level detected by RT-qPCR and (B) protein level detected by Western blot in primary cells treated with vehicle control or CX-4945 for 48 hrs. (C-H) Expression of *GLUT1* in (C, E, G) mRNA level detected by RT-qPCR and (D, F, H) protein level detected by Western blot in primary cells. Patient 1 treated with vehicle control, imatinib (1 μM) or ponatinib (50 nM), CX-4945 (3 μM), and a combination for 48 hrs. Patient 2 treated with vehicle control, imatinib (4 μM), CX-4945 (5 μM), and a combination for 48 hrs. Patient 3 treated with vehicle control, imatinib (2 μM) or ponatinib (80 nM), CX-4945 (5 μM), and the combination for 48 hrs. * $P < .05$, ** $P < .01$, *** $P < .001$.

Figure. S17

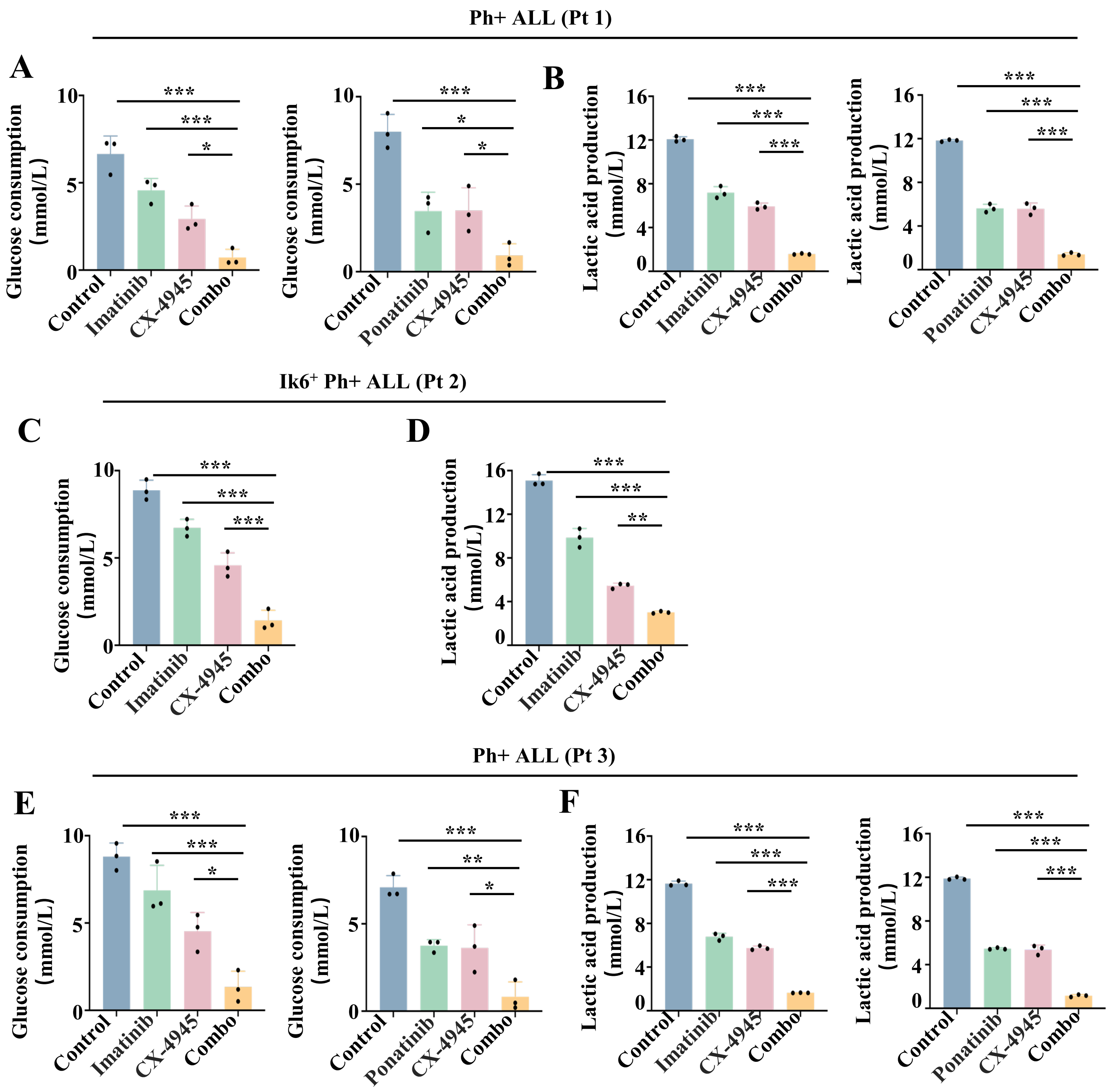

Fig. S17 Effect of imatinib or ponatinib with CX-4945 on aerobic glycolysis in primary cells. (A-F) Comparison of (A, C, E) glucose consumption and (B, D, F) lactic acid production in primary cells. Patient 1 treated with vehicle control, imatinib (1 μM) or ponatinib (50 nM), CX-4945 (3 μM), and a combination for 48 hrs. Patient 2 treated with vehicle control, imatinib (4 μM), CX-4945 (5 μM), and a combination for 48 hrs. Patient 3 treated with vehicle control, imatinib (2 μM) or ponatinib (80 nM), CX-4945 (5 μM), and the combination for 48 hrs. * $P < .05$, ** $P < .01$, *** $P < .001$.

Figure. S18

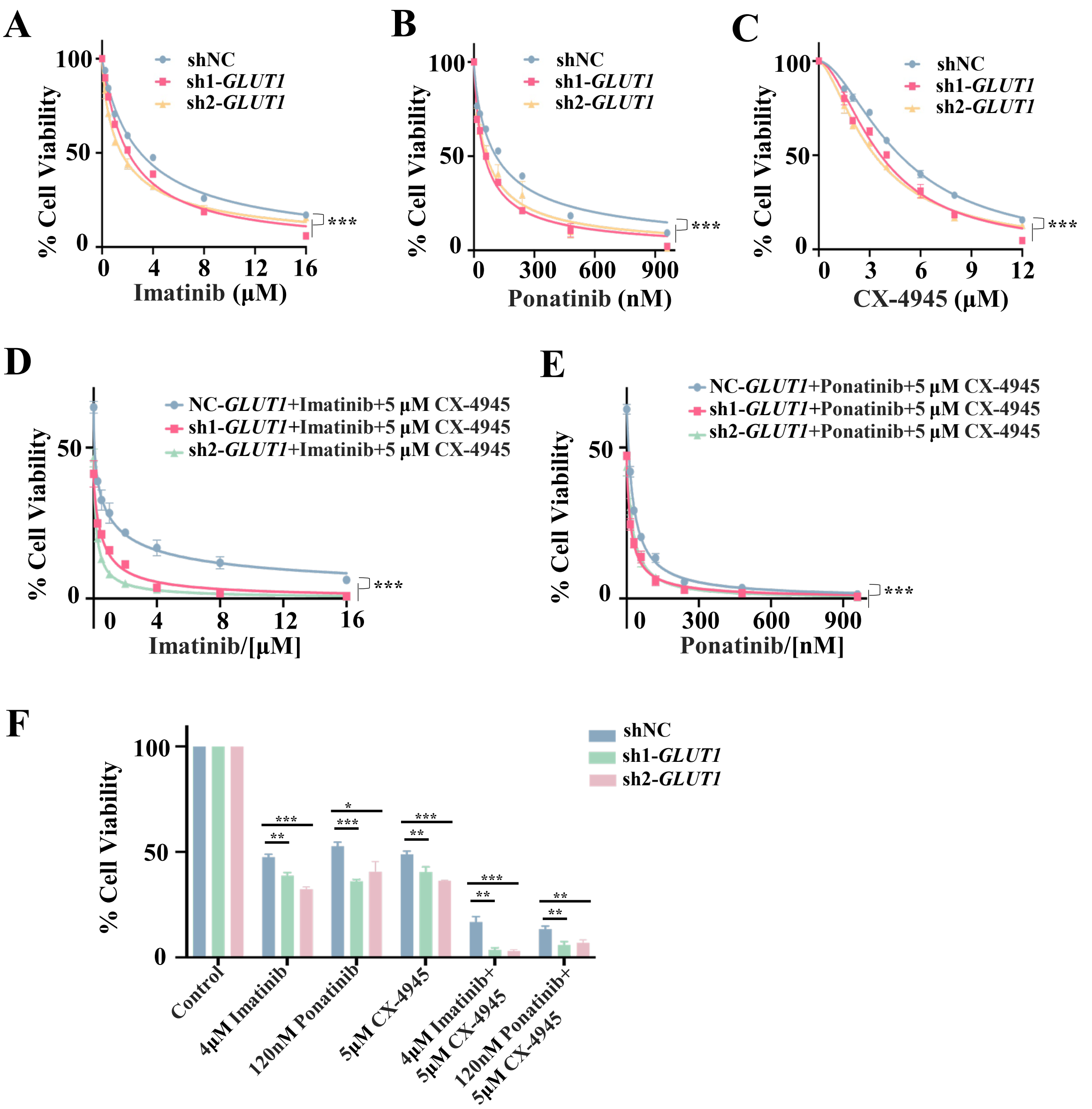

Fig. S18 Effect of imatinib or ponatinib with CX-4945 on SUP-B15 cells with sh*GLUT1*. (A-C) Effect of (A) imatinib, (B) ponatinib, or (C) CX-4945 on the cell proliferation in SUP-B15 cells with sh*GLUT1* *vs.* shNC. The cells are stably transduced with the shRNAs and treated with the indicated doses of the drugs for 48 hrs. (D-E) Synergistic effect of (D) imatinib or (E) ponatinib with CX-4945 on the cell viability for the synergy of two drugs in SUP-B15 cells with sh*GLUT1* (SUP-B15-sh*GLUT1*) *vs* shNC. Cells were treated with the indicated drugs for 48 hrs. (F) Comparison of the synergistic analysis of imatinib or ponatinib with CX-4945 cell viability in SUP-B15-sh*GLUT1* *vs.* SUP-B15-shNC. * $P < .05$, ** $P < .01$, *** $P < .001$.

Figure. S19

A

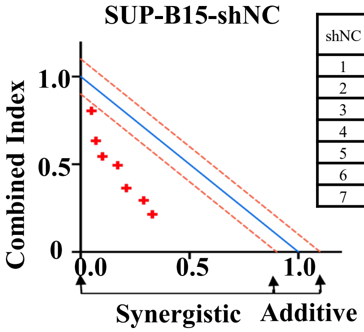

shNC	Imatinib (μM)	CX-4945 (μM)	CI
1	0.25	5	0.85
2	0.5		0.69
3	1		0.64
4	2		0.66
5	4		0.57
6	8		0.58
7	16		0.54

B

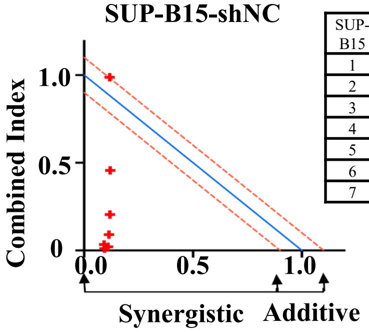

SUP-B15	Ponatinib (nM)	CX-4945 (μM)	CI
1	15	5	1.10
2	30		0.57
3	60		0.32
4	120		0.20
5	240		0.12
6	480		0.13
7	960		0.10

C

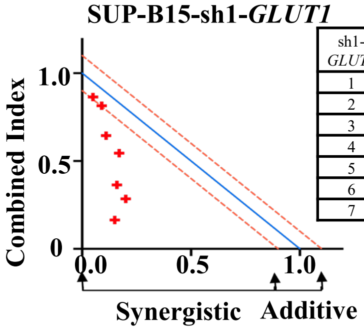

sh1-*GLUT1*	Imatinib (μM)	CX4945 (μM)	CI
1	0.25	5	0.90
2	0.5		0.90
3	1		0.76
4	2		0.71
5	4		0.52
6	8		0.47
7	16		0.31

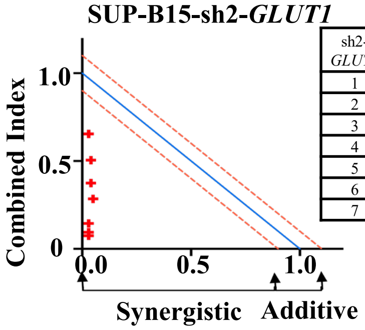

sh2-*GLUT1*	Imatinib (μM)	CX4945 (μM)	CI
1	0.25	5	0.68
2	0.5		0.54
3	1		0.41
4	2		0.34
5	4		0.17
6	8		0.12
7	16		0.10

D

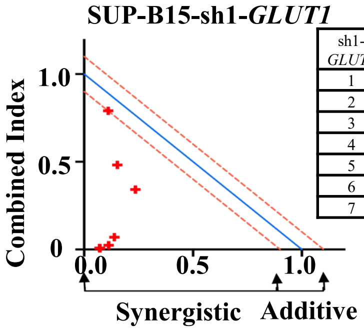

sh1-*GLUT1*	Ponatinib (nM)	CX-4945 (μM)	CI
1	15	5	0.90
2	30		0.63
3	60		0.57
4	120		0.20
5	240		0.13
6	480		0.08
7	960		0.07

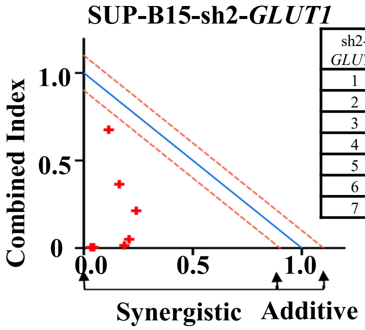

sh2-*GLUT1*	Ponatinib (nM)	CX-4945 (μM)	CI
1	15	5	0.79
2	30		0.52
3	60		0.45
4	120		0.25
5	240		0.20
6	480		0.05
7	960		0.03

Fig. S19 The synergistic effects of imatinib or ponatinib with CX-4945 on SUP-B15 cells. (A-B) CalcuSyn analysis of synergy of the (A) imatinib or (B) ponatinib with CX-4945 on cell viability in SUP-B15 cells transduced with scramble shRNA(SUP-B15-shNC). (C-D) CalcuSyn analysis of synergy of the (C) imatinib or (D) ponatinib with CX-4945 on cell viability in SUP-B15-sh*GLUT1*. Cells were treated with the indicated drugs for 48 hrs.

Figure. S20

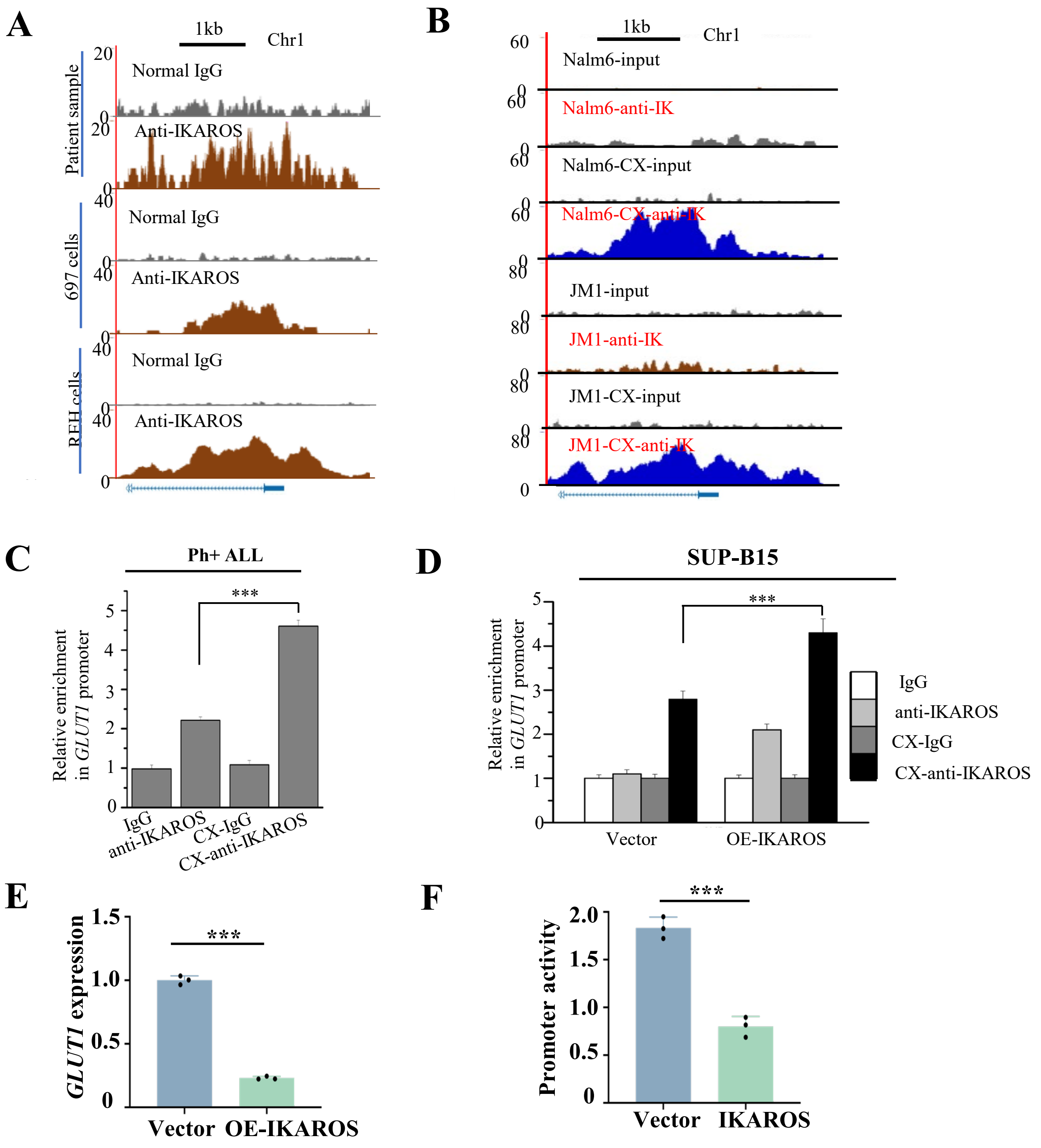

Fig. S20 IKAROS binding peaks in the promoter region of the *GLUT1* gene identified by ChIP-seq. (A) IKAROS binding peaks in the promoter region of the *GLUT1* gene in patient samples and B-ALL cell lines, 697 and REH. (B) Effect of CX-4945 (CX) on the IKAROS (IK) binding enrichment in the promoter region of Nalm6 and JM1 B-ALL cells. Cells were treated with 10 µM CX-4945 for 48 hrs, and the nucleus of the cells was prepared for the ChIP-seq assay. (C) Effect of CX-4945 on IKAROS binding enrichment in the promoter of the *GLUT1* gene of Ph+ ALL primary cells without Ik6 by qChIP assay. Cells were treated with 10 µM CX-4945 for 48 hrs. (D) The effect of CX-4945 on IKAROS binding enrichment in the promoter of the *GLUT1* gene in SUP-B15 cells by qChIP assay was identified by qChIP. Cells were treated with 10 µM CX-4945 for 48 hrs. (E-F) Effect of IKAROS overexpression (OE-IKAROS) on (E) *GLUT1* expression in mRNA level by RT-qPCR in SUP-B15 cells, and (F) promoter activity identified by luciferase reporter assay. *** $P < 0.001$.

Figure. S21

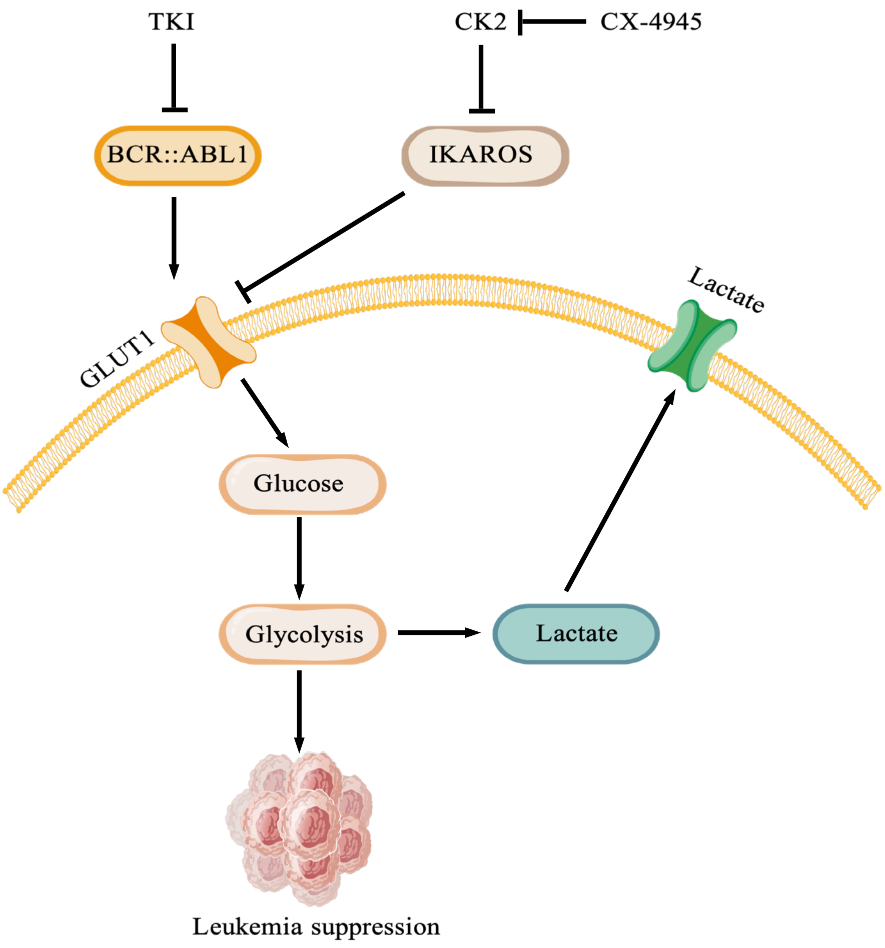

Fig. S21 New mechanism model to overcome the TKI resistance via restoring IKAROS transcriptional repression of *GLUT1* and further suppression of aerobic glycolysis in Ph+ ALL.
